# Impact of respiratory motion on ^18^F‐FDG PET radiomics stability: Clinical evaluation with a digital PET scanner

**DOI:** 10.1002/acm2.14200

**Published:** 2023-11-08

**Authors:** Yu‐Hung Chen, Kuo‐Yi Kan, Shu‐Hsin Liu, Hsin‐Hon Lin, Kun‐Han Lue

**Affiliations:** ^1^ Department of Nuclear Medicine Hualien Tzu Chi Hospital Buddhist Tzu Chi Medical Foundation Hualien Taiwan; ^2^ School of Medicine College of Medicine Tzu Chi University Hualien Taiwan; ^3^ Department of Medical Imaging and Radiological Sciences Tzu Chi University of Science and Technology Hualien Taiwan; ^4^ Department of Nuclear Medicine Fu Jen Catholic University Hospital New Taipei City Taiwan; ^5^ Department of Medical Imaging and Radiological Sciences College of Medicine Chang Gung University Taoyuan Taiwan; ^6^ Department of Nuclear Medicine Chang Gung Memorial Hospital Linkou Taiwan

**Keywords:** ^18^F‐FDG, digital PET, radiomics, respiratory motion, stability

## Abstract

**Purpose:**

^18^F‐FDG PET quantitative features are susceptible to respiratory motion. However, studies using clinical patient data to explore the impact of respiratory motion on ^18^F‐FDG PET radiomic features are limited. In this study, we investigated the impact of respiratory motion on radiomics stability with clinical ^18^F‐FDG PET images using a data‐driven gating (DDG) algorithm on the digital PET scanner.

**Materials and Methods:**

A total of 101 patients who underwent oncological ^18^F‐FDG PET scans were retrospectively included. A DDG algorithm combined with a motion compensation technique was used to extract the PET images with respiratory motion correction. ^18^F‐FDG‐avid lesions from the thorax to the upper abdomen were analyzed on the non‐DDG and DDG PET images. The lesions were segmented with a 40% threshold of the maximum standardized uptake. A total of 725 radiomic features were computed from the segmented lesions, including first‐order, shape, texture, and wavelet features. The intraclass correlation coefficient (ICC) and coefficient of variation (COV) were calculated to evaluate feature stability. An ICC above 0.9 and a COV below 5% were considered high stability.

**Results:**

In total, 168 lesions with and without respiratory motion correction were analyzed. Our results indicated that most ^18^F‐FDG PET radiomic features are sensitive to respiratory motion. Overall, only 27 out of 725 (3.72%) radiomic features were identified as highly stable, including one from the first‐order features (entropy), one from the shape features (sphericity), four from the gray‐level co‐occurrence matrix features (normalized and unnormalized inverse difference moment, joint entropy, and sum entropy), one from the gray‐level run‐length matrix features (run entropy), and 20 from the wavelet filter‐based features.

**Conclusion:**

Respiratory motion has a significant impact on ^18^F‐FDG PET radiomics stability. The highly stable features identified in our study may serve as potential candidates for further applications, such as machine learning modeling.

## 1 INTRODUCTION

Respiratory motion is a source of image degradation in positron emission tomography (PET), potentially leading to lower detection rates for malignancies.^1^ Respiratory motion‐induced image degradation may occur in the thorax and upper abdomen,^2^ particularly near the diaphragm.^3^ Because of the long acquisition duration of PET, the negative effects of respiratory motion on the interpretation of PET images are inevitable and considerable.^4^ The intensity, size, and precise location of a lesion on images are influenced by respiratory motion, which may mislead the diagnosis. Therefore, the correction of respiratory motion is essential in PET images.

^18^F‐fludeoxyglucose (^18^F‐FDG) PET is widely used to assess glucose metabolic information in primary tumors, lymph nodes, or distant metastases in oncological patients.^5^ With the advent of novel digital PET scanner, the lesion detection capability shows an improvement, owing to the higher sensitivity and better spatial resolution compared to the traditional analog PET scanner.^6^, ^7^ However, the adverse impact of respiratory motion is more pronounced for PET scanners with a higher spatial resolution, highlighting the importance of motion correction by the digital system.^8^ The data‐driven gating (DDG) technique, which provides respiratory‐gated PET images for motion correction, has been commercially developed.^9^, ^10^ The DDG algorithm can be implemented to compensate for the negative effects of respiratory motion and refine the increased resolution of digital PET scanners.

Radiomics denotes the high‐throughput extraction of numerous quantitative metrics from medical images.^11^ ^18^F‐FDG PET radiomics has been widely explored for building diagnostic, predictive, and prognostic models to pursue personalized management in oncology studies.^12^ However, the quantification of ^18^F‐FDG PET images is susceptible to respiratory motion.^13^ Motion, especially respiratory motion, can introduce a blurring effect in the PET images.^14^ These motion‐blurred images may lead to large biases in lesion uptake intensity and volume estimates.^15^ Several studies have been conducted to evaluate the variability in radiomic features for respiratory motion in ^18^F‐FDG PET images.^16^, ^17^, ^18^, ^19^, ^20^ However, these studies were based on small patient groups^16^, ^18^, ^20^ or phantoms^17^, ^19^ and showed contradictory results. Faist et al.^18^ and Fuaki et al.^20^ evaluated 141 and 46 features in 20 patients with lung cancer, respectively. Both reported that most features (131/141 and 39/46) can be used interchangeably between non‐gating PET and DDG PET. On the contrary, Oliver et al.^16^ examined the 56 features in 23 lung cancer patients and found significantly different image feature values caused by the respiratory motion. In the phantom studies, Xu et al.^17^ and Hosseini et al.^19^ showed that the robustness of features is considerably affected by respiratory motion.

The interpretation of previous findings needs further evaluation in a larger clinical patient cohort. Furthermore, although ^18^F‐FDG PET radiomics have been vigorously tested for their diagnostic and prognostic roles, the robustness of the image features should also be investigated.^21^ Radiomic features are considerable in number, and hence, ensuring their stability against respiratory motion is desirable before clinical implementation. With this premise, this study aimed to investigate the impact of respiratory motion on the stability of radiomic features with clinical ^18^F‐FDG PET images using DDG respiratory motion correction on a digital PET scanner.

## 2 MATERIALS AND METHODS

### 2.1 Study population

Patients diagnosed with oncological pathologies, who underwent ^18^F‐FDG PET with DDG respiratory motion compensation between August 2020 and July 2021, were retrospectively analyzed. Patients were referred for initial staging or restaging diseases after one or more therapy courses. The ^18^F‐FDG PET images were interpreted in consensus by two experienced nuclear medicine physicians. All included patients had at least one ^18^F‐FDG‐avid lesion (primary or metastatic lesion) located in the thorax or upper abdomen. The institutional review board and research ethics committee approved this study. Given the retrospective nature of this study, the need for informed consent was waived.

### 2.2 Image acquisition

All patients fasted for at least 6 h before the administration of ^18^F‐FDG (400 MBq) and had blood glucose levels under 200 mg/dL. Whole‐body scans were conducted from the mid‐thigh to the vertex using a four‐ring GE Discovery MI PET/CT scanner (GE Healthcare, Milwaukee, USA). The Discovery MI PET scanner is equipped with a digital silicon photomultiplier (SiPM) readout and time‐of‐flight (TOF) technology. A DDG algorithm (MotionFree, GE Healthcare, Milwaukee, USA), combined with a motion correction technique (Q.Static, GE Healthcare, Milwaukee, USA), was used to acquire the PET images with respiratory motion correction.^22^ The algorithm was set with default parameters, including a phase offset of 30%, a phase window width of 50%, and an R‐threshold of 15.

All PET scans were performed 45–60 min after the intravenous injection of the radiotracer and were acquired in three‐dimensional mode with 150 s per table position. The CT scans were conducted with a tube voltage of 120 kV and an automated tube current ranging between 15 and 180 mA to correct for the attenuation of the PET emission data. It was suggested that the CT tube current has no significant effect on the ^18^F‐FDG uptake values or lesion size in reconstructed PET images.^23^ All PET images were reconstructed with a fixed protocol using the Bayesian penalized likelihood reconstruction algorithm^24^ Q.Clear (*β* = 550). The reconstructed PET images of all patients had a matrix size of 256 × 256, a pixel size of 2.73 mm × 2.73 mm, and a slice thickness of 2.79 mm. Two PET image datasets with and without DDG respiratory motion correction were generated for each patient.

### 2.3 Radiomic analysis

The motion‐corrected and uncorrected PET images were analyzed by the same reviewer to reduce interobserver variability. The results were subsequently confirmed by another experienced nuclear medicine physician. ^18^F‐FDG‐avid lesions were automatically segmented using a fixed threshold of 40% of the maximum standardized uptake value (SUV), which exhibited superior interobserver reproducibility for texture analysis.^25^ The segmented volumes were used to define the metabolic tumor volume (MTV). Each segmented lesion contained at least 64 voxels, recommended for calculating textural features.^26^ The segmented lesions were classified into three groups, including all lesions, large lesions (MTV ≥3 cm^3^),^27^ and small lesions (MTV <3 cm^3^). All PET images were analyzed using PMOD image processing software version 4.204 (PMOD Technologies Ltd., Zurich, Switzerland).

A total of 725 radiomic features, including 18 first‐order, 14 shape‐, 61 texture‐, and 632 wavelet‐based features, were extracted from the segmented lesions using a fixed bin width of 0.25 SUV.^28^ The textural features were extracted from gray‐level co‐occurrence matrix (GLCM, *n* = 24), gray‐level run‐length matrix (GLRLM, *n* = 16), gray‐level size‐zone matrix (GLSZM, *n* = 16), and neighboring gray‐tone difference matrix (NGTDM, *n* = 5). Wavelet filters were applied to transform PET images into eight decompositions (coif1 wavelet) using low‐frequency (L) and high‐frequency (H) filtering functions in each of the three dimensions (x, y, z).

For each patient, two sets of ^18^F‐FDG PET radiomic data were generated: with and without DDG respiratory motion correction. The Pyradiomics (Harvard Medical School, Boston, USA)^29^ open‐source software package version 3.0.1 was used to calculate the radiomic features in compliance with the definitions described by the Imaging Biomarker Standardization Initiative.^30^ Table S1 presents a list of computed radiomic features in this study.

### 2.4 Statistical and data analysis

Continuous variables are expressed as median values and interquartile ranges (IQR). The intraclass correlation coefficient (ICC) was used to measure the reliability of measurements for radiomic features between PET images with and without DDG motion compensation. The obtained ICC was computed using a single‐measurement, absolute‐agreement, two‐way random‐effects model.^31^ ICC was calculated as follows:

(1) $$ICC=\frac{MS_{R}-MS_{E}}{MS_{R}+\left(K-1\right)MS_{E}+\frac{k}{n}\left(MS_{C}-MS_{E}\right)}$$

where *MS_R_*, *MS_E_*, *MS_C_*, *k*, and *n* denote the mean square for rows, mean square for error, mean square for columns, number of measurements, and number of subjects, respectively.

All features were divided into four categories in terms of ICC values. ICC less than 0.5, between 0.5 and 0.75, between 0.75 and 0.9, and greater than 0.90 were indicative of poor, moderate, good, and excellent reliability, respectively.

The calculation of the coefficient of variation (COV) was used to determine the reproducibility of the measurements.^32^ The COV was estimated for radiomic features from the PET images with and without DDG motion correction using the following equation:

(2) $$COV\left(\%\right)=\frac{SD}{Mean}\times 100$$

where *SD* and *Mean* represent the standard deviation and average of the duplicate measurements, respectively.^33^ All features were divided into four categories based on the COV values^34^: below 5%, between 5% and 10%, between 10% and 20%, and greater than 20%, indicative of excellent, good, moderate, and poor reproducibility, respectively.

All statistical analyses, including ICC and COV calculations, were performed using MedCalc version 22.009 (MedCalc Software, Ostend, Belgium).

## 3 RESULTS

### 3.1 Patient characteristics

**FIGURE 1:**
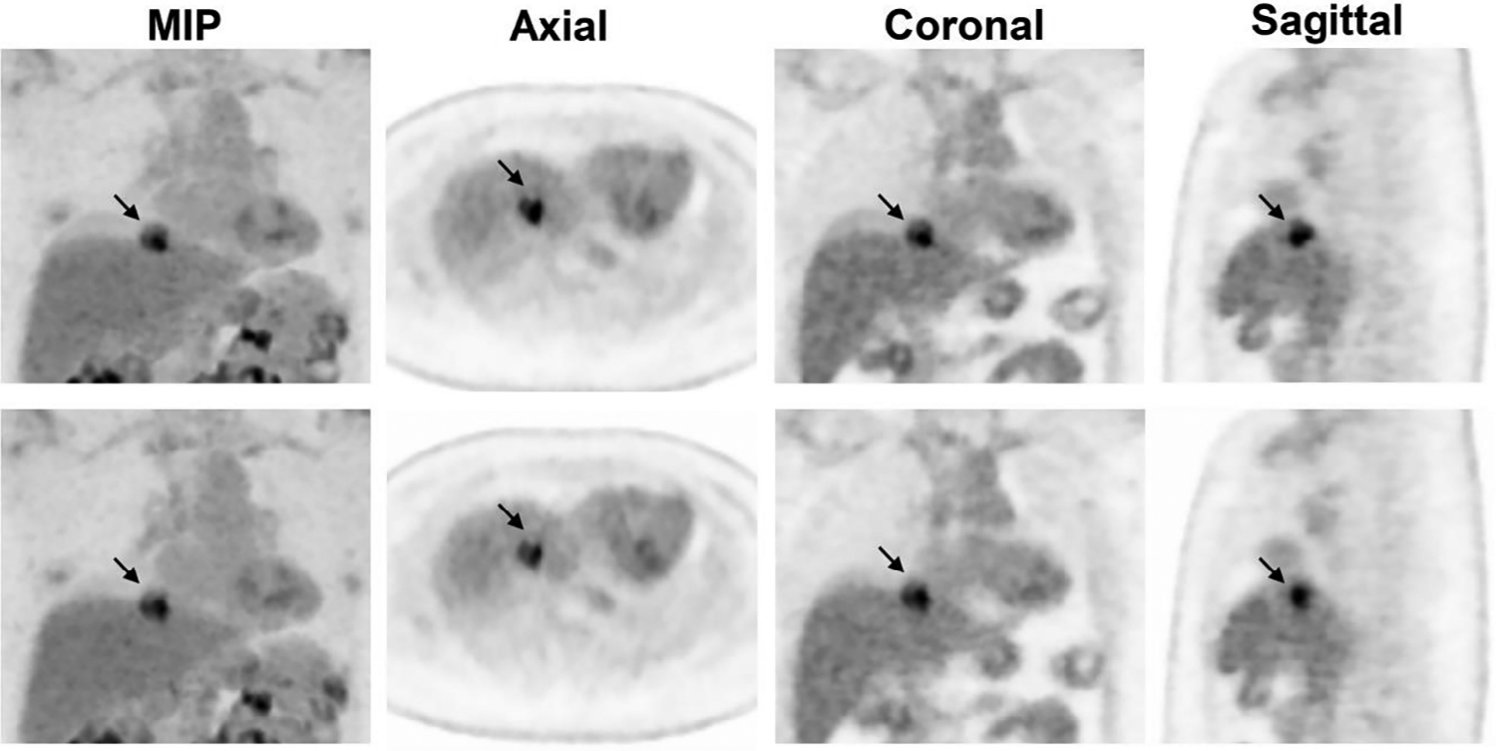
Representative PET images demonstrate alterations in lesion shape and heterogeneity with (top row) and without (bottom row) respiratory motion correction. MIP, maximum intensity projection.

**FIGURE 2:**
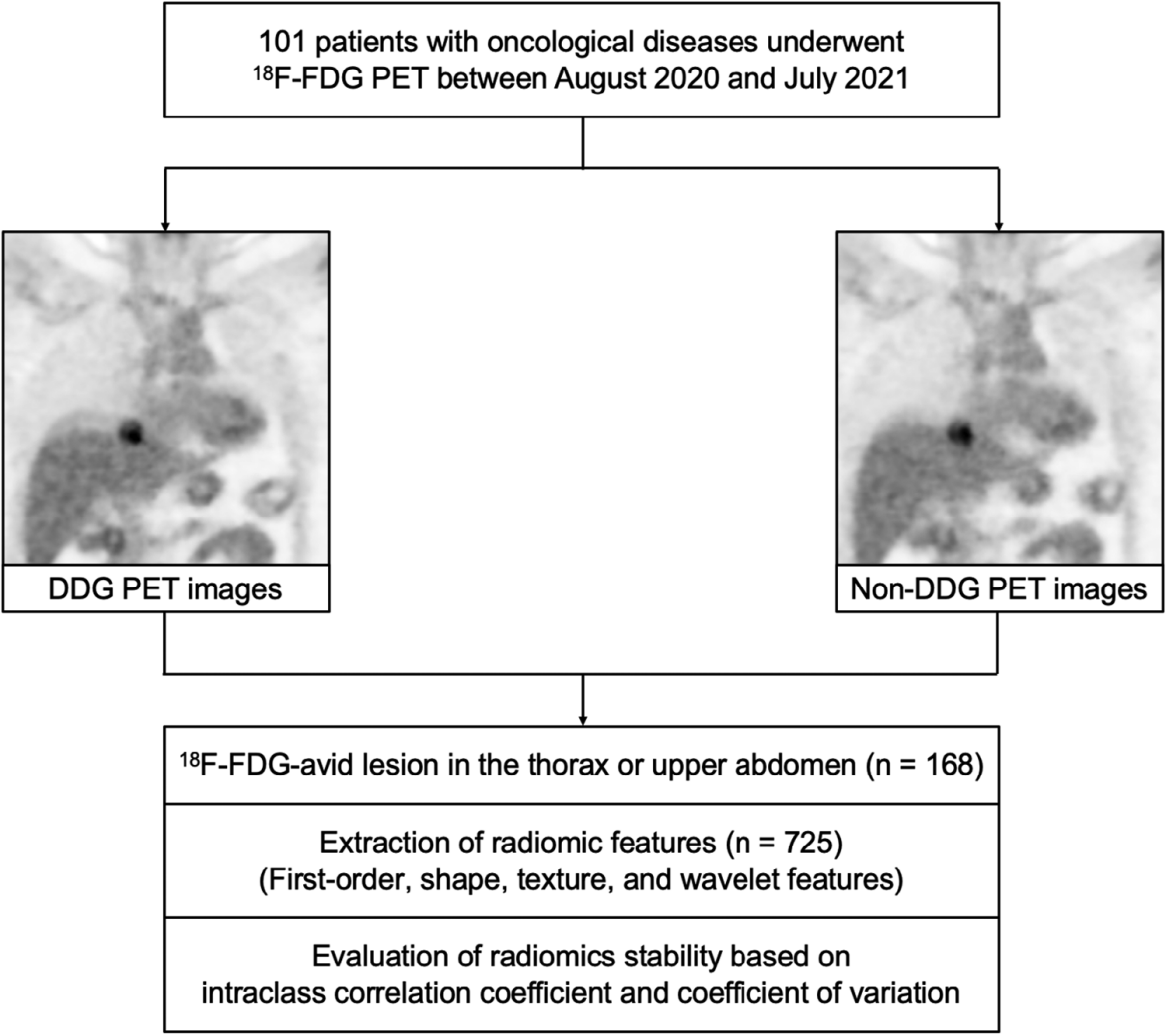
Flowchart of the study design. DDG, data‐driven gating.

A total of 101 patients with different oncological diseases undergoing ^18^F‐FDG PET were enrolled in the study. Malignancies were pathologically confirmed in all the patients. The demographic information is presented in Table 1. The median BMI for the entire cohort was 22.7 kg/m^2^ (IQR, 6.33 kg/m^2^). A total of 168 lesions were analyzed for computing radiomic features. Among them, 99 lesions were located in the thorax, and 69 were in the upper abdomen. According to the DDG PET, the median MTV of the analyzed lesions was 3.40 cm^3^ (IQR, 6.58 cm^3^) using 40% thresholding of the maximum SUV. Of these, 88 lesions had an MTV above 3 cm^3^. Figure 1 shows the representative PET images with visual alterations in the lesion shape and heterogeneity, with and without DDG respiratory compensation. Figure 2 presents a flowchart of the study design.

**TABLE 1:** General characteristics of patients.

Characteristic	Value
Gender, *n* (%)	
Female	43 (42.6%)
Male	58 (57.4%)
Age, median (IQR), years	63 (13.3)
Heigh, median (IQR), m	1.61 (0.11)
Weight, median (IQR), kg	58 (17.3)
Type of primary disease, *n* (%)	
Lung cancer	38 (37.6%)
Colon cancer	17 (16.8%)
Head and neck cancer	16 (15.8%)
Esophageal cancer	14 (13.9%)
Breast cancer	6 (5.9%)
Urogenital cancer	4 (4.0%)
Lymphoma	4 (4.0%)
Pancreatic cancer	2 (2.0%)

Abbreviation: IQR, interquartile range.

### 3.2 Radiomic features reliability

**FIGURE 3:**
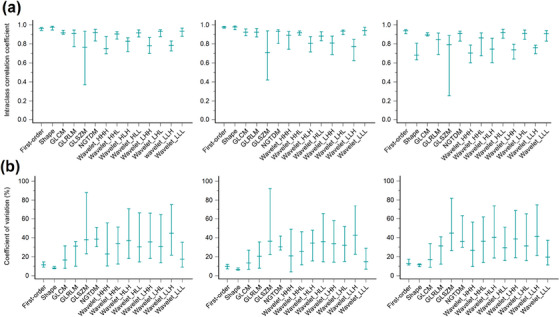
The distributions of (a) intraclass correlation coefficient (ICC) and (b) coefficient of variation (COV) for each radiomic feature type in all lesions (first column), large lesions (second column), and small lesions (third column). The middle horizontal lines represent the median, and the top and bottom horizontal lines represent the interquartile range in the box plots. GLCM, gray‐level co‐occurrence matrix; GLRLM, gray‐level run‐length matrix; GLSZM, gray‐level size‐zone matrix; NGTDM, neighboring gray‐tone difference matrix. Wavelet filtering yielded eight decompositions, applying either a High (H) or a Low (L) pass filter in each x‐, y‐, and z‐dimension.

The distributions of ICC (median and IQR) for each radiomic feature type are presented in Figure 3a. The median values of ICC were 0.876 (IQR, 0.170), 0.891 (IQR, 0.161), and 0.823 (IQR, 0.227) in the groups of all lesions, large lesions, and small lesions, respectively. Considering all lesions, the shape features showed the highest ICC (median 0.974, IQR 0.040), and the wavelet_HHH features had the lowest ICC (median 0.750, IQR 0.183). For large lesions, the shape features also showed the highest ICC (median 0.978, IQR 0.038), while the GLSZM features had the lowest ICC (median 0.710, IQR 0.517). Among small lesions, the original first‐order features exhibited the highest ICC (median 0.940, IQR 0.042), while the shape features showed the lowest ICC (median 0.681, IQR 0.175).

**FIGURE 4:**
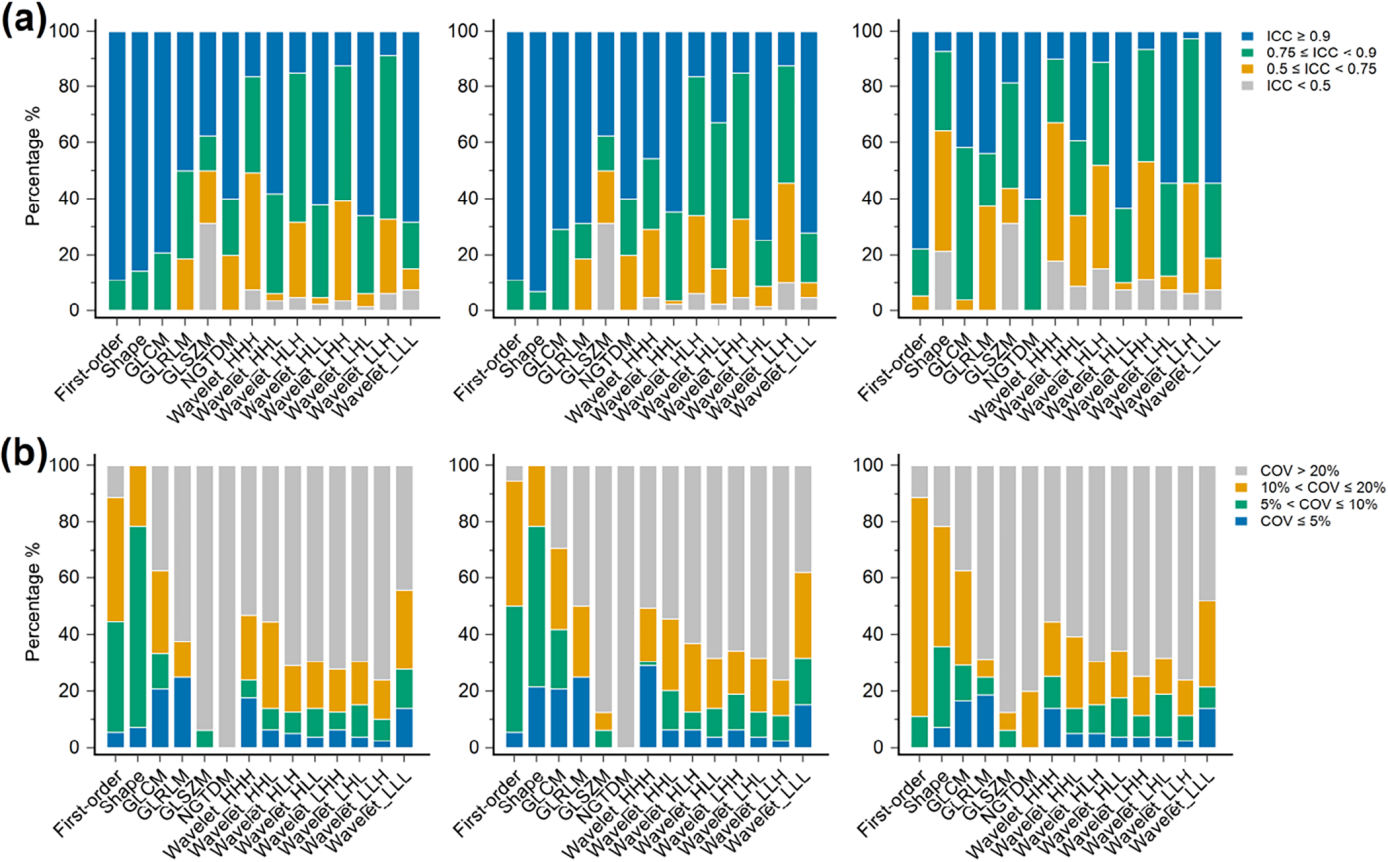
The percentages of radiomic features classified based on (a) intraclass correlation coefficient (ICC) and (b) coefficient of variation (COV) for each feature type in all lesions (first column), large lesions (second column), and small lesions (third column). GLCM, gray‐level co‐occurrence matrix; GLRLM, gray‐level run‐length matrix; GLSZM, gray‐level size‐zone matrix; NGTDM, neighboring gray‐tone difference matrix. Wavelet filtering yielded eight decompositions, applying either a High (H) or a Low (L) pass filter in each x‐, y‐, and z‐dimension.

Based on the ICC, the percentages of radiomic features classified for each feature type are shown in Figure 4a. The number of reliable radiomic features, identified by the ICC above 0.9, was 307 (42.3%), 330 (45.5%), and 229 (31.6%) for all lesions, large lesions, and small lesions, respectively. The original first‐order features had the highest percentage of an ICC above 0.9 in all lesions (16/18, 89.9%) and small lesions (14/18, 77.8%) groups, respectively. For large lesions, the shape features exhibited the highest percentage of an ICC above 0.9 (13/14, 92.7%). The wavelet_LLH features showed the lowest percentage with an ICC above 0.9 in all three groups (7/79, 8.86% for all lesions, 10/79, 12.7% for large lesions, and 2/79, 2.53% for small lesions, respectively). The GLSZM features demonstrated the worst performance in terms of reliability, with the highest percentage (5/16, 31.3%) having an ICC of less than 0.5 in all three groups.

### 3.3 Radiomic features reproducibility

The distributions of COV (median and IQR) for each radiomic feature type are presented in Figure 3b. The median values of COV were 28.66% (IQR, 45.65%), 27.65% (IQR, 38.03%), and 30.37% (IQR, 44.77%) in the groups of all lesions, large lesions, and small lesions, respectively. The shape features maintained the smallest COV in all three groups (median 8.047%, IQR 2.423% for all lesions; median 6.463%, IQR 2.255% for large lesions; median 11.54%, IQR 2.827% for small lesions). The wavelet_LLH features had the greatest COV in the groups of all lesions (median 44.74%, IQR 53.85%) and large lesions (median 42.63%, IQR 51.50%). The GLSZM features showed the greatest COV (median 44.76%, IQR 55.56%) for the small lesions.

According to COV, the percentages of radiomic features classified for each feature type are shown in Figure 4b. The number of reproducible radiomic features identified by COV below 5% was 58 (8.0%), 71 (9.8%), and 49 (6.8%) for the groups of all lesions, large lesions, and small lesions, respectively. The GLRLM features had the highest proportion with a COV below 5% in all lesions (4/16, 25.0%) and small lesions (3/16, 18.8%%), respectively. For large lesions, the wavelet_HHH features had the highest percentage of COV below 5% (23/79, 29.1%). The GLSZM and NGTDM features showed the lowest percentage with a COV below 5% in all three groups (no features meet the criteria). The NGTDM features exhibited the worst performance in terms of reproducibility for the groups of all lesions and large lesions, as each feature had a COV greater than 20% (5/5, 100%). The GLSZM features revealed the worst performance for small lesions, with the highest percentage (14/16, 87.5%) having a COV greater than 20%.

### 3.4 Radiomic features with high and low stabilities

Overall, 27/725 (3.72%) radiomic features were identified to have high stability according to both reliability and reproducibility (ICC ≥ 0.9 and COV ≤ 5%). Among these features, one was from the first‐order features (entropy), one from the shape features (sphericity), four from the GLCM features (normalized and unnormalized inverse difference moment, joint entropy, and sum entropy), one from the GLRLM features (run entropy), and 20 from the wavelet filter‐based features. Regarding the low stability (ICC < 0.5 and COV > 20%), a total of 33/725 (4.55%) radiomic features were identified. Of these, five were from the GLSZM features, and the remaining 28 were from the wavelet filter‐based features. A detailed list is available in Table 2. A subgroup analysis for radiomic features with high and low stabilities in the large and small lesion sizes is listed in Tables S2 and S3, respectively. Heatmaps of radiomic features based on ICC and COV stratified by lesion size are shown in Figures S1 and S2, respectively.

**TABLE 2:** Radiomic features with high and low stabilities against respiratory motion.

Group	Type	Description
High stability(ICC ≥ 0.9 and COV ≤ 5%)	First‐order	Entropy
	Shape	Sphericity
	GLCM	Inverse Difference Moment Normalized Inverse Difference Normalized Joint Entropy Sum Entropy
	GLRLM	Run Entropy
	Wavelet	HHH_GLRLM_Run Entropy HHH_GLRLM_Run Length NonUniformity Normalized HHH_GLRLM_Run Percentage HHH_GLRLM_Short Run Emphasis HHL_GLCM_Inverse Difference HHL_GLCM_Inverse Difference Moment HHL_GLRLM_Run Entropy HLL_GLCM_Inverse Difference Moment Normalized HLL_GLCM_Inverse Difference Normalized HLL_GLRLM_Run Entropy LHL_GLCM_Inverse Difference Moment Normalized LHL_GLCM_Inverse Difference Normalized LHL_GLRLM_Run Entropy LLL_First‐order_Entropy LLL_GLCM_Inverse Difference Moment Normalized LLL_GLCM_Inverse Difference Normalized LLL_GLCM_Joint Entropy LLL_GLCM_Sum Entropy LLL_GLRLM_Run Entropy LLL_GLSZM_Zone Entropy
Low stability (ICC < 0.5 and COV > 20%)	GLSZM	Large Area Emphasis Large Area High Gray Level Emphasis Large Area Low Gray Level Emphasis Small Area Low Gray Level Emphasis Zone Variance
	Wavelet	HHH_First‐order_Skewness HHH_GLCM_Cluster Shade HHH_GLSZM_Size Zone Non Uniformity Normalized HHH_GLSZM_Small AreaLow GrayLevel Emphasis HHL_GLSZM_Size Zone Non Uniformity Normalized HHL_GLSZM_Small Area Emphasis HHL_GLSZM_Small AreaLow GrayLevel Emphasis HLH_GLCM_Cluster Shade HLH_GLSZM_Size Zone Non Uniformity Normalized HLH_GLSZM_Small Area Emphasis HLH_GLSZM_Small Area Low Gray Level Emphasis HLL_GLSZM_Small Area Low Gray Level Emphasis HLL_NGTDM_Busyness LHH_GLSZM_Size Zone NonUniformity Normalized LHH_GLSZM_Small Area Emphasis LHH_GLSZM_Small Area Low Gray Level Emphasis LHL_GLSZM_Small Area Low Gray Level Emphasis LLH_GLSZM_Large Area Emphasis LLH_GLSZM_Large Area Low GrayLevel Emphasis LLH_GLSZM_Small Area Low Gray Level Emphasis LLH_GLSZM_Zone Variance LLH_NGTDM_Busyness LLL_GLSZM_Large Area Emphasis LLL_GLSZM_Large Area High GrayLevel Emphasis LLL_GLSZM_Large Area Low Gray Level Emphasis LLL_GLSZM_Low Gray Level Zone Emphasis LLL_GLSZM_Small Area Low Gray Level Emphasis LLL_GLSZM_Zone Variance

Abbreviations: COV, coefficient of variation; GLCM, gray‐level co‐occurrence matrix; GLRLM, gray‐level run‐length matrix; GLSZM, gray‐level size‐zone matrix; ICC, intraclass correlation coefficient; NGTDM, neighboring gray‐tone difference matrix. Wavelet filtering yielded eight decompositions, applying either a High (H) or a Low (L) pass filter in each x‐, y‐, and z‐dimension.

### 3.5 Stability of radiomic features based on lesion size

**FIGURE 5:**
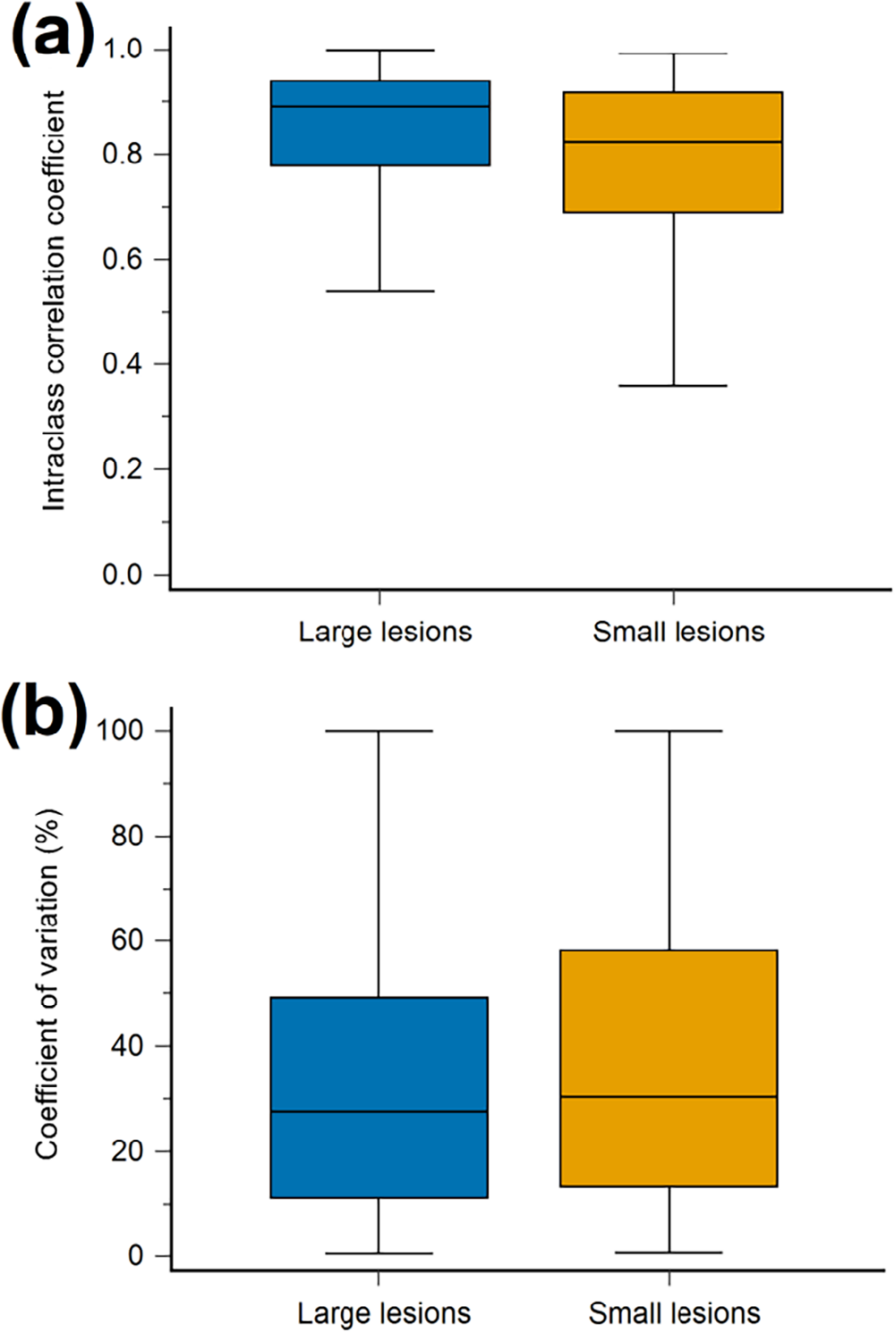
Boxplots of the (a) intraclass correlation coefficient (ICC) and (b) coefficient of variation (COV) for the groups of large lesions and small lesions. Large lesions were defined as a metabolic tumor volume (MTV) of ≥3 cm^3^, and small lesions were defined as MTV < 3 cm^3^. The middle horizontal lines represent the median, the box represents the interquartile range, and the top and bottom horizontal lines represent the highest and lowest values.

**FIGURE 6:**
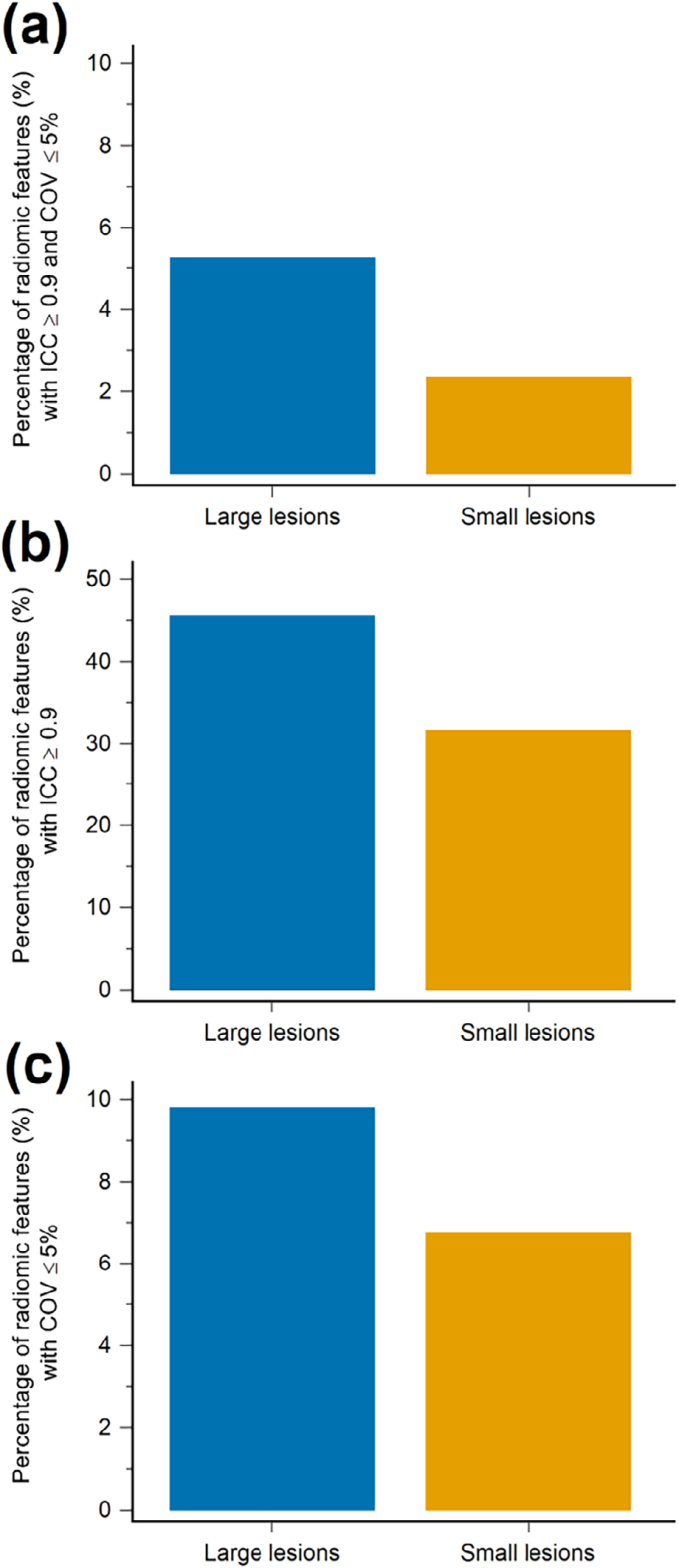
Bar graphs of percentage of radiomic features with (a) intraclass correlation coefficient (ICC) ≥0.9 and coefficient of variation (COV) ≤5%, with (b) ICC ≥ 0.9, and with (c) COV ≤ 5% stratified by lesion size. Large lesions were defined as a metabolic tumor volume (MTV) of ≥3 cm^3^), and small lesions were defined as MTV < 3 cm^3^.

When stratified by MTV into large (≥3 cm^3^) and small lesion (<3 cm^3^) size groups, the median ICC decreased from 0.891 to 0.823 for large and small lesions (Figure 5a). The median COV increased from 27.65% to 30.37% with decreasing lesion size (Figure 5b). An overall increase in the percentage of stable radiomic features was observed as the lesion size increased, based on ICC ≥ 0.9 or COV ≤ 5% criteria. In the large lesion group, 5.24% (38/725) of the radiomic features exhibited both ICC ≥ 0.9 and COV ≤ 5%, whereas in the small lesion group, the percentage was 2.34% (17/725) (Figure 6a). Radiomic features with ICC ≥ 0.9 accounted for 45.52% (330/725) in the large lesion group and 31.59% (229/725) in the small lesion group (Figure 6b). Regarding COV ≤ 5%, 9.79% (71/725) of the radiomic features met this criterion in the large lesion group, while in the small lesion group, the percentage was 6.76% (49/725) (Figure 6c).

## 4 DISCUSSION

The present study investigated the impact of respiratory motion on ^18^F‐FDG radiomics stability in patients with oncological diseases. Using the DDG algorithm with the digital PET scanner, our finding indicated that respiratory motion considerably impacted the stability of radiomic features. To date, several studies have reported the influence of motion on radiomic analysis.^16^, ^17^, ^18^, ^19^, ^20^ However, the previous findings were contradictory. Additionally, the results of previous studies were mainly in terms of small patient groups (*n* < 30)^16^, ^18^, ^20^ and in‐house developed phantoms.^17^, ^19^ Our study examined the stability of 725 radiomic features based on 168 lesions derived from clinical patients (*n* = 101), complementing the findings of previously published research.

In our study, 27 out of 725 radiomic features exhibited high stability in terms of ICC and COV. ICC and COV were chosen as evaluation metrics because these two coefficients are conceptually distinct.^21^, ^35^ The radiomic features with high stability identified in this study have demonstrated robustness against the motion in previous research,^16^, ^17^, ^19^ except wavelet‐based filtering features. Fewer studies have primarily investigated the impact of motion on wavelet‐based features in ^18^F‐FDG PET radiomics. Our study identified 20 out of 632 wavelet features as stable. Overall, this finding is consistent with that of Oliveira et al., who reported that wavelet features were highly dependent on respiratory motion in lung cancer patients,^36^ and Vuong et al., who found that wavelet features had lower stability on a 4D‐gated PET/MR dataset.^37^ Interestingly, the robust wavelet features did not depend on images with emphasized heterogeneity (high‐pass filtering) or smoothed images (low‐pass filtering) but rather on the texture characteristics per se with different wavelet subbands (Table 2). Our findings may be helpful in providing stable radiomic features for establishing reliable prediction models.

Further, our study conducted a subgroup analysis based on the lesion sizes. Grootjans et al.^27^ investigated the influence of respiratory motion on the quantification of textural parameters in ^18^F‐FDG PET images. The study preselected lesions with a volume above 3 cm^3^ and indicated that entropy was not significantly affected by respiratory motion, which is in line with our findings. In addition, the results of a phantom study showed that the robustness of the radiomic features is lesion size dependent,^19^ which is in accordance with our results. Both respiratory motion and lesion size affect the radiomic stability. The number of highly stable radiomic features was higher in the large‐lesion group than in the small‐lesion group (Table S2 vs. Table S3). Respiratory motion has a more pronounced effect on small lesions than on large ones. Regarding the unstable radiomic features, the lowest stability was observed for features based on GLSZM in our study. Previous studies have reported that GLSZM‐based features are the most susceptible to variability.^38^ Another study has suggested that the GLSZM‐based features exhibited the lowest reproducibility against the synergistic impact of motion, acquisition, and reconstruction parameters,^19^ which appear to be compatible with our study. The present study enhances the previous findings by providing data on radiomics with low stability against respiratory motion.

In the phantom studies, Xu et al.^17^ showed that 79/487 (16%) of features were identified to be highly stable to respiratory motion, and Hosseini et al.^19^ revealed that 51/174 (29%) of radiomic features were robust against motion. In a clinical study, Oliver et al.^16^ reported that 9/56 (16%) features had the least variability between static and respiratory‐gated PET images. Our study found that only 27/725 (3.72%) features exhibited high stability, which agrees with previous reports. Our results identified fewer stable features compared to those studies mentioned above. A possible explanation for this result is that we adopted both ICC and COV to evaluate radiomics with high stability. Conversely, Faist et al.^18^ reported that 131/141 (93%) features were not affected by respiratory movements, and Fuaki et al.^20^ demonstrated 39/46 (85%) image features had adequate reproducibility between non‐gating and DDG PET based on clinical studies. The results of those studies might seem to contradict previous findings. Because we found that only 8.0% of features showed high reproducibility (COV below 5%), this inconsistency may be attributed to the fact that COV was not examined in both their studies. Another possible explanation may lie in the small size of the patient group in their studies.

The present findings contribute to the field's understanding of the impact of respiratory motion on ^18^F‐FDG PET radiomics stability. The results confirm that most radiomic features are vulnerable to respiratory motion, as reported previously,^16^, ^17^, ^19^ and reproducibility and reliability should be a concern. PET radiomic features are considered crucial biomarkers in the field of oncology.^39^ However, biomarkers should be reproducible and reliable for clinical implementation. Therefore, it is desirable to ensure radiomics stability for identifying features as suitable candidates to generalize diagnostic, predictive, and prognostic models in the era of personalized medicine. Our study paves the way for future research, enabling model generalization in machine learning using identified radiomic features that exhibit high stability.

We acknowledge that this study has several limitations. First, the current evaluation was based on a heterogeneous study population. The impact of DDG on different oncological diseases may vary. Second, the stability of radiomics can also be affected by the image acquisition and reconstruction protocols.^40^, ^41^ Further research should be undertaken to investigate the synergistic impact of acquisition and reconstruction parameters in conjunction with respiratory motion on the stability of radiomics in a prospective clinical study. Third, the DDG algorithm was set with default parameters provided by the manufacturer without optimization in our center. Fourth, a threshold of 64 voxels for each segmented lesion was adopted to calculate the texture features in the radiomic analysis. Further studies are required to establish a standardized threshold for the minimum number of voxels required in quantitative PET radiomics. Fifth, because the ground truth values of radiomic features are unknown in clinical PET images, future studies are recommended to precisely assess the extent of the impact of respiratory motion on radiomic stability by utilizing Monte Carlo simulations with an anthropomorphic digital phantom.^42^, ^43^ Finally, although respiratory motion influenced the radiomic features, whether it significantly impacts the clinical value (diagnostic, predictive, and prognostic) needs further validation. Future studies in large and homogeneous patient populations correlating to clinical outcomes are warranted.

## 5 CONCLUSION

In summary, we investigated the impact of respiratory motion on ^18^F‐FDG PET radiomics stability. The study demonstrated that respiratory motion reduced the reliability and reproducibility of radiomic features derived from clinical PET images. Only 27 out of 725 (3.72%) radiomic features demonstrated high stability. Radiomic features with high stability can be suitable candidates for further applications, such as machine learning modeling. Features with low stability should be omitted while applying radiomics in clinical applications.

## AUTHOR CONTRIBUTIONS

Conceptualization and study design: Yu‐Hung Chen and Kun‐Han Lue. Data collection: Kuo‐Yi Kan and Shu‐Shin Liu. Image analysis: Yu‐Hung Chen, Shu‐Shin Liu, and Kun‐Han Lue. Statistical and data analysis: Yu‐Hung Chen, Kuo‐Yi Kan, Hsin‐Hon Lin, and Kun‐Han Lue. Manuscript writing and revision: Yu‐Hung Chen and Kun‐Han Lue. All authors read and approved the final manuscript.

## CONFLICT OF INTEREST STATEMENT

All authors declare that they have no conflicts of interest.

## ETHICS STATEMENT

The institutional review board and research ethics committee approved this study (No. IRB110‐136‐B).

## Supporting information

Supporting‐Information

Supporting‐Information

## References

[1] Manber R , Thielemans K , Hutton BF , et al. Clinical impact of respiratory motion correction in simultaneous PET/MR, using a joint PET/MR Predictive Motion Model. J Nucl Med. 2018;59(9):1467‐1473. 29523630 10.2967/jnumed.117.191460 PMC6126439

[2] Pépin A , Daouk J , Bailly P , Hapdey S , Meyer ME . Management of respiratory motion in PET/computed tomography: the state of the art. Nucl Med Commun. 2014;35(2):113‐122. 24352107 10.1097/MNM.0000000000000048 PMC3868022

[3] Hamill JJ , Bosmans G , Dekker A . Respiratory‐gated CT as a tool for the simulation of breathing artifacts in PET and PET/CT. Med Phys. 2008;35(2):576‐585. 18383679 10.1118/1.2829875

[4] Bai W , Brady M . Motion correction and attenuation correction for respiratory gated PET images. IEEE Trans Med Imaging. 2011;30(2):351‐365. 20875967 10.1109/TMI.2010.2078514

[5] Boellaard R , Delgado‐Bolton R , Oyen WJ , et al. FDG PET/CT: EANM procedure guidelines for tumour imaging: version 2.0. Eur J Nucl Med Mol Imaging. 2015;42(2):328‐354. 25452219 10.1007/s00259-014-2961-x PMC4315529

[6] Delcroix O , Bourhis D , Keromnes N , et al. Assessment of image quality and lesion detectability with digital PET/CT system. Front Med (Lausanne). 2021;8:629096. 33693016 10.3389/fmed.2021.629096 PMC7937710

[7] Surti S , Viswanath V , Daube‐Witherspoon ME , Conti M , Casey ME , Karp JS . Benefit of improved performance with State‐of‐the Art Digital PET/CT for lesion detection in Oncology. J Nucl Med. 2020;61(11):1684‐1690. 32198313 10.2967/jnumed.120.242305 PMC9364891

[8] Polycarpou I , Tsoumpas C , King AP , Marsden PK . Impact of respiratory motion correction and spatial resolution on lesion detection in PET: a simulation study based on real MR dynamic data. Phys Med Biol. 2014;59(3):697‐713. 24442386 10.1088/0031-9155/59/3/697

[9] Aide N , Lasnon C , Desmonts C , Armstrong IS , Walker MD , McGowan DR . Advances in PET/CT technology: an update. Semin Nucl Med. 2022;52(3):286‐301. 34823841 10.1053/j.semnuclmed.2021.10.005

[10] Lamare F , Bousse A , Thielemans K , et al. PET respiratory motion correction: quo vadis? Phys Med Biol. 2022;67(3):1‐27. 10.1088/1361-6560/ac43fc 34915465

[11] Hatt M , Krizsan AK , Rahmim A , et al. Joint EANM/SNMMI guideline on radiomics in nuclear medicine : jointly supported by the EANM Physics Committee and the SNMMI Physics, Instrumentation and Data Sciences Council. Eur J Nucl Med Mol Imaging. 2023;50(2):352‐375. 36326868 10.1007/s00259-022-06001-6 PMC9816255

[12] Limkin EJ , Sun R , Dercle L , et al. Promises and challenges for the implementation of computational medical imaging (radiomics) in oncology. Ann Oncol. 2017;28(6):1191‐1206. 28168275 10.1093/annonc/mdx034

[13] Liu C , Pierce LA 2nd , Alessio AM , Kinahan PE . The impact of respiratory motion on tumor quantification and delineation in static PET/CT imaging. Phys Med Biol. 2009;54(24):7345‐7362. 19926910 10.1088/0031-9155/54/24/007 PMC2895622

[14] Soret M , Bacharach SL , Buvat I . Partial‐volume effect in PET tumor imaging. J Nucl Med. 2007;48(6):932‐945. 17504879 10.2967/jnumed.106.035774

[15] Erdi YE , Nehmeh SA , Pan T , et al. The CT motion quantitation of lung lesions and its impact on PET‐measured SUVs. J Nucl Med. 2004;45(8):1287‐1292. 15299050

[16] Oliver JA , Budzevich M , Zhang GG , Dilling TJ , Latifi K , Moros EG . Variability of image features computed from conventional and respiratory‐gated PET/CT images of lung cancer. Transl Oncol. 2015;8(6):524‐534. 26692535 10.1016/j.tranon.2015.11.013 PMC4700295

[17] Xu H , Lv W , Zhang H , Ma J , Zhao P , Lu L . Evaluation and optimization of radiomics features stability to respiratory motion in ^18^F‐FDG 3D PET imaging. Med Phys. 2021;48(9):5165‐5178. 34085282 10.1002/mp.15022

[18] Faist D , Jreige M , Oreiller V , et al. Reproducibility of lung cancer radiomics features extracted from data‐driven respiratory gating and free‐breathing flow imaging in ^18^F‐FDG PET/CT. Eur J Hybrid Imaging. 2022;6(1):33. 36309636 10.1186/s41824-022-00153-2 PMC9617997

[19] Hosseini SA , Shiri I , Hajianfar G , et al. Synergistic impact of motion and acquisition/reconstruction parameters on ^18^F‐FDG PET radiomic features in non‐small cell lung cancer: phantom and clinical studies. Med Phys. 2022;49(6):3783‐3796. 35338722 10.1002/mp.15615 PMC9322423

[20] Fukai S , Daisaki H , Ishiyama M , et al. Reproducibility of the principal component analysis (PCA)‐based data‐driven respiratory gating on texture features in non‐small cell lung cancer patients with ^18^F‐FDG PET/CT. J Appl Clin Med Phys. 2023;24(5):e13967. 36943700 10.1002/acm2.13967 PMC10161026

[21] Zwanenburg A . Radiomics in nuclear medicine: robustness, reproducibility, standardization, and how to avoid data analysis traps and replication crisis. Eur J Nucl Med Mol Imaging. 2019;46(13):2638‐2655. 31240330 10.1007/s00259-019-04391-8

[22] Walker MD , Morgan AJ , Bradley KM , McGowan DR . Evaluation of data‐driven respiratory gating waveforms for clinical PET imaging. EJNMMI Res. 2019;9(1):1. 30607651 10.1186/s13550-018-0470-9 PMC6318161

[23] Kamel E , Hany TF , Burger C , et al. CT vs 68Ge attenuation correction in a combined PET/CT system: evaluation of the effect of lowering the CT tube current. Eur J Nucl Med Mol Imaging. 2002;29(3):346‐350. 12002709 10.1007/s00259-001-0698-9

[24] Messerli M , Stolzmann P , Egger‐Sigg M , et al. Impact of a Bayesian penalized likelihood reconstruction algorithm on image quality in novel digital PET/CT: clinical implications for the assessment of lung tumors. EJNMMI Phys. 2018;5(1):27. 30255439 10.1186/s40658-018-0223-x PMC6156690

[25] Bashir U , Azad G , Siddique MM , et al. The effects of segmentation algorithms on the measurement of ^18^F‐FDG PET texture parameters in non‐small cell lung cancer. EJNMMI Res. 2017;7(1):60. 28748524 10.1186/s13550-017-0310-3 PMC5529305

[26] Orlhac F , Nioche C , Klyuzhin I , Rahmim A , Buvat I . Radiomics in PET imaging: a practical guide for newcomers. PET Clin. 2021;16(4):597‐612. 34537132 10.1016/j.cpet.2021.06.007

[27] Grootjans W , Tixier F , van der Vos CS , et al. The impact of optimal respiratory gating and image noise on evaluation of intratumor heterogeneity on ^18^F‐FDG PET Imaging of Lung Cancer. J Nucl Med. 2016;57(11):1692‐1698. 27283931 10.2967/jnumed.116.173112

[28] Pfaehler E , van Sluis J , Merema BBJ , et al. Experimental multicenter and multivendor evaluation of the performance of PET radiomic features using 3‐Dimensionally printed phantom inserts. J Nucl Med. 2020;61(3):469‐476. 31420497 10.2967/jnumed.119.229724 PMC7067530

[29] van Griethuysen JJM , Fedorov A , Parmar C , et al. Computational radiomics system to decode the radiographic phenotype. Cancer Res. 2017;77(21):e104‐e107. 29092951 10.1158/0008-5472.CAN-17-0339 PMC5672828

[30] Zwanenburg A , Vallières M , Abdalah MA , et al. The image biomarker standardization initiative: standardized quantitative radiomics for high‐throughput image‐based phenotyping. Radiology. 2020;295(2):328‐338. 32154773 10.1148/radiol.2020191145 PMC7193906

[31] Koo TK , Li MY . A guideline of selecting and reporting intraclass correlation coefficients for reliability research. J Chiropr Med. 2016;15(2):155‐163. 27330520 10.1016/j.jcm.2016.02.012 PMC4913118

[32] Tian L . Inferences on the common coefficient of variation. Stat Med. 2005;24(14):2213‐2220. 15803444 10.1002/sim.2088

[33] Synek V . Evaluation of the standard deviation from duplicate results. Accredit Qual Assur. 2008;13(6):335‐337.

[34] Shiri I , Rahmim A , Ghaffarian P , Geramifar P , Abdollahi H , Bitarafan‐Rajabi A . The impact of image reconstruction settings on ^18^F‐FDG PET radiomic features: multi‐scanner phantom and patient studies. Eur Radiol. 2017;27(11):4498‐4509. 28567548 10.1007/s00330-017-4859-z

[35] Kottner J , Audigé L , Brorson S , et al. Guidelines for Reporting Reliability and Agreement Studies (GRRAS) were proposed. J Clin Epidemiol. 2011;64(1):96‐106. 21130355 10.1016/j.jclinepi.2010.03.002

[36] Oliveira C , Amstutz F , Vuong D , et al. Preselection of robust radiomic features does not improve outcome modelling in non‐small cell lung cancer based on clinical routine FDG‐PET imaging. EJNMMI Res. 2021;11(1):79. 34417899 10.1186/s13550-021-00809-3 PMC8380219

[37] Vuong D , Tanadini‐Lang S , Huellner MW , et al. Interchangeability of radiomic features between ^18^F‐FDG PET/CT and ^18^F‐FDG PET/MR. Med Phys. 2019;46(4):1677‐1685. 30714158 10.1002/mp.13422

[38] Sollini M , Cozzi L , Antunovic L , Chiti A , Kirienko M . PET radiomics in NSCLC: state of the art and a proposal for harmonization of methodology. Sci Rep. 2017;7(1):358. 28336974 10.1038/s41598-017-00426-y PMC5428425

[39] Lee JW , Lee SM . Radiomics in oncological PET/CT: clinical applications. Nucl Med Mol Imaging. 2018;52(3):170‐189. 29942396 10.1007/s13139-017-0500-y PMC5995782

[40] Hatt M , Cheze Le Rest C , Antonorsi N , et al. Radiomics in PET/CT: current status and future AI‐based evolutions. Semin Nucl Med. 2021;51(2):126‐133. 33509369 10.1053/j.semnuclmed.2020.09.002

[41] Lovinfosse P , Visvikis D , Hustinx R , Hatt M . FDG PET radiomics: a review of the methodological aspects. Clin Transl Imaging. 2018;6(5):379‐391.

[42] Vauclin S , Michel C , Buvat I , et al. Monte‐Carlo simulations of clinically realistic respiratory gated ^18^F‐FDG PET: application to lesion detectability and volume measurements. Comput Methods Programs Biomed. 2015;118(1):84‐93. 25459525 10.1016/j.cmpb.2014.10.003

[43] Yang F , Young LA , Johnson PB . Quantitative radiomics: validating image textural features for oncological PET in lung cancer. Radiother Oncol. 2018;129(2):209‐217. 30279049 10.1016/j.radonc.2018.09.009

