# Supplementary material for: Impact of respiratory motion on 18F‐FDG PET radiomics stability: Clinical evaluation with a digital PET scanner
Source: J Appl Clin Med Phys. 2023 Nov 8;24(12):e14200. doi: 10.1002/acm2.14200 (PMC10691638; doi:10.1002/acm2.14200)
Supplement: Supplementary file 1 — Supporting‐Information [file ACM2-24-e14200-s001.pdf]

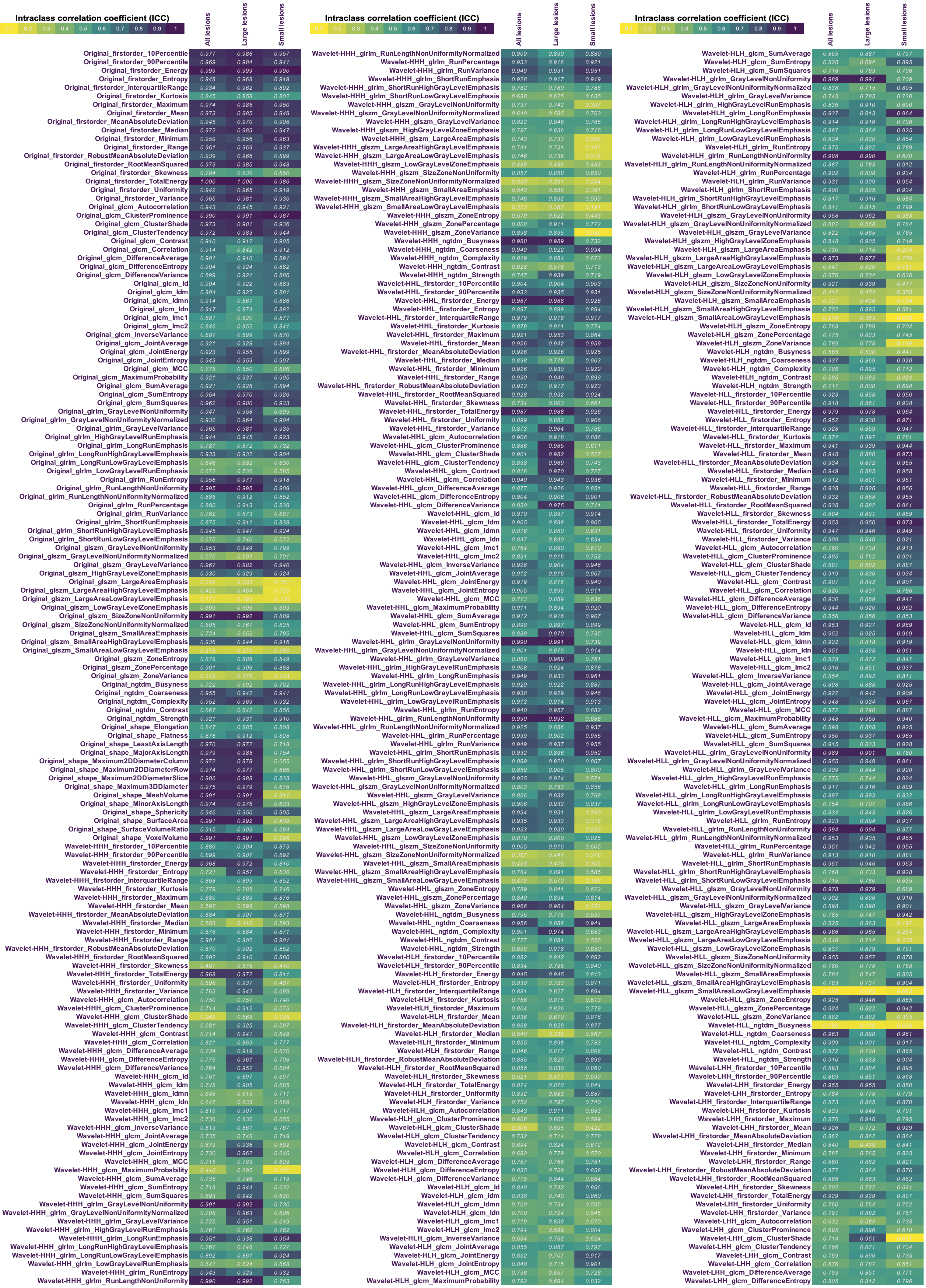

FIGURE S1 Heatmap of radiomic features based on intraclass correlation coefficient (ICC) divided by lesion size.

Intraclass correlation coefficient (ICC)

0.1 0.2 0.3 0.4 0.5 0.6 0.7 0.8 0.9 1

Feature	All lesions	Large lesions	Small lesions
Wavelet-LHH_glcm_DifferenceVariance	0.758	0.906	0.691
Wavelet-LHH_glcm_Id	0.798	0.805	0.788
Wavelet-LHH_glcm_Idm	0.796	0.809	0.784
Wavelet-LHH_glcm_Idmn	0.679	0.688	0.651
Wavelet-LHH_glcm_Idn	0.687	0.712	0.582
Wavelet-LHH_glcm_Imc1	0.715	0.856	0.580
Wavelet-LHH_glcm_Imc2	0.704	0.621	0.689
Wavelet-LHH_glcm_InverseVariance	0.737	0.766	0.718
Wavelet-LHH_glcm_JointAverage	0.711	0.688	0.750
Wavelet-LHH_glcm_JointEnergy	0.768	0.764	0.769
Wavelet-LHH_glcm_JointEntropy	0.793	0.779	0.795
Wavelet-LHH_glcm_MCC	0.653	0.622	0.636
Wavelet-LHH_glcm_MaximumProbability	0.700	0.754	0.681
Wavelet-LHH_glcm_SumAverage	0.711	0.688	0.750
Wavelet-LHH_glcm_SumEntropy	0.780	0.765	0.785
Wavelet-LHH_glcm_SumSquares	0.770	0.882	0.737
Wavelet-LHH_glrlm_GrayLevelNonUniformity	0.989	0.991	0.706
Wavelet-LHH_glrlm_GrayLevelNonUniformityNormalized	0.779	0.735	0.790
Wavelet-LHH_glrlm_GrayLevelVariance	0.777	0.881	0.733
Wavelet-LHH_glrlm_HighGrayLevelRunEmphasis	0.654	0.605	0.753
Wavelet-LHH_glrlm_LongRunEmphasis	0.936	0.930	0.935
Wavelet-LHH_glrlm_LongRunHighGrayLevelEmphasis	0.724	0.710	0.706
Wavelet-LHH_glrlm_LongRunLowGrayLevelEmphasis	0.826	0.760	0.922
Wavelet-LHH_glrlm_LowGrayLevelRunEmphasis	0.640	0.594	0.704
Wavelet-LHH_glrlm_RunEntropy	0.867	0.894	0.756
Wavelet-LHH_glrlm_RunLengthNonUniformity	0.988	0.991	0.664
Wavelet-LHH_glrlm_RunLengthNonUniformityNormalized	0.866	0.818	0.860
Wavelet-LHH_glrlm_RunPercentage	0.892	0.850	0.890
Wavelet-LHH_glrlm_RunVariance	0.941	0.928	0.950
Wavelet-LHH_glrlm_ShortRunEmphasis	0.887	0.846	0.892
Wavelet-LHH_glrlm_ShortRunHighGrayLevelEmphasis	0.687	0.633	0.763
Wavelet-LHH_glrlm_ShortRunLowGrayLevelEmphasis	0.574	0.564	0.559
Wavelet-LHH_glszm_GrayLevelNonUniformity	0.919	0.923	0.455
Wavelet-LHH_glszm_GrayLevelNonUniformityNormalized	0.710	0.698	0.719
Wavelet-LHH_glszm_GrayLevelVariance	0.791	0.859	0.698
Wavelet-LHH_glszm_HighGrayLevelZoneEmphasis	0.700	0.669	0.780
Wavelet-LHH_glszm_LargeAreaEmphasis	0.897	0.895	0.137
Wavelet-LHH_glszm_LargeAreaHighGrayLevelEmphasis	0.893	0.888	0.158
Wavelet-LHH_glszm_LargeAreaLowGrayLevelEmphasis	0.527	0.500	0.119
Wavelet-LHH_glszm_LowGrayLevelZoneEmphasis	0.653	0.665	0.637
Wavelet-LHH_glszm_SizeZoneNonUniformity	0.831	0.826	0.659
Wavelet-LHH_glszm_SizeZoneNonUniformityNormalized	0.361	0.334	0.381
Wavelet-LHH_glszm_SmallAreaEmphasis	0.470	0.417	0.468
Wavelet-LHH_glszm_SmallAreaHighGrayLevelEmphasis	0.700	0.682	0.736
Wavelet-LHH_glszm_SmallAreaLowGrayLevelEmphasis	0.269	0.323	0.161
Wavelet-LHH_glszm_ZoneEntropy	0.677	0.678	0.631
Wavelet-LHH_glszm_ZonePercentage	0.809	0.772	0.796
Wavelet-LHH_glszm_ZoneVariance	0.939	0.936	0.319
Wavelet-LHH_ngtdm_Busyness	0.577	0.533	0.509
Wavelet-LHH_ngtdm_Coarseness	0.948	0.886	0.935
Wavelet-LHH_ngtdm_Complexity	0.846	0.927	0.681
Wavelet-LHH_ngtdm_Contrast	0.636	0.649	0.609
Wavelet-LHH_ngtdm_Strength	0.671	0.855	0.636
Wavelet-LHL_firstorder_10Percentile	0.945	0.930	0.951
Wavelet-LHL_firstorder_90Percentile	0.921	0.930	0.914
Wavelet-LHL_firstorder_Energy	0.981	0.981	0.973
Wavelet-LHL_firstorder_Entropy	0.959	0.938	0.977
Wavelet-LHL_firstorder_InterquartileRange	0.923	0.909	0.923
Wavelet-LHL_firstorder_Kurtosis	0.893	0.878	0.883
Wavelet-LHL_firstorder_Maximum	0.933	0.936	0.928
Wavelet-LHL_firstorder_Mean	0.949	0.934	0.958
Wavelet-LHL_firstorder_MeanAbsoluteDeviation	0.936	0.927	0.937
Wavelet-LHL_firstorder_Median	0.944	0.944	0.942
Wavelet-LHL_firstorder_Minimum	0.937	0.922	0.955
Wavelet-LHL_firstorder_Range	0.941	0.937	0.948
Wavelet-LHL_firstorder_RobustMeanAbsoluteDeviation	0.932	0.920	0.932
Wavelet-LHL_firstorder_RootMeanSquared	0.941	0.933	0.944
Wavelet-LHL_firstorder_Skewness	0.889	0.901	0.863
Wavelet-LHL_firstorder_TotalEnergy	0.971	0.971	0.945
Wavelet-LHL_firstorder_Uniformity	0.955	0.948	0.962
Wavelet-LHL_firstorder_Variance	0.882	0.940	0.862
Wavelet-LHL_glcm_Autocorrelation	0.948	0.951	0.946
Wavelet-LHL_glcm_ClusterProminence	0.803	0.957	0.752
Wavelet-LHL_glcm_ClusterShade	0.876	0.968	0.846
Wavelet-LHL_glcm_ClusterTendency	0.878	0.931	0.859
Wavelet-LHL_glcm_Contrast	0.865	0.938	0.852
Wavelet-LHL_glcm_Correlation	0.856	0.849	0.853
Wavelet-LHL_glcm_DifferenceAverage	0.931	0.920	0.931
Wavelet-LHL_glcm_DifferenceEntropy	0.949	0.933	0.961
Wavelet-LHL_glcm_DifferenceVariance	0.904	0.957	0.875
Wavelet-LHL_glcm_Id	0.957	0.932	0.974
Wavelet-LHL_glcm_Idm	0.956	0.928	0.975
Wavelet-LHL_glcm_Idmn	0.903	0.842	0.899
Wavelet-LHL_glcm_Idn	0.951	0.916	0.957
Wavelet-LHL_glcm_Imc1	0.873	0.859	0.836
Wavelet-LHL_glcm_Imc2	0.930	0.899	0.937
Wavelet-LHL_glcm_InverseVariance	0.876	0.928	0.798
Wavelet-LHL_glcm_JointAverage	0.933	0.925	0.944
Wavelet-LHL_glcm_JointEnergy	0.938	0.943	0.932
Wavelet-LHL_glcm_JointEntropy	0.954	0.938	0.977
Wavelet-LHL_glcm_MCC	0.849	0.822	0.837
Wavelet-LHL_glcm_MaximumProbability	0.955	0.955	0.955
Wavelet-LHL_glcm_SumAverage	0.933	0.925	0.944
Wavelet-LHL_glcm_SumEntropy	0.960	0.945	0.977
Wavelet-LHL_glcm_SumSquares	0.876	0.933	0.859
Wavelet-LHL_glrlm_GrayLevelNonUniformity	0.989	0.990	0.826
Wavelet-LHL_glrlm_GrayLevelNonUniformityNormalized	0.959	0.947	0.971
Wavelet-LHL_glrlm_GrayLevelVariance	0.884	0.942	0.862
Wavelet-LHL_glrlm_HighGrayLevelRunEmphasis	0.944	0.950	0.937
Wavelet-LHL_glrlm_LongRunEmphasis	0.914	0.917	0.879
Wavelet-LHL_glrlm_LongRunHighGrayLevelEmphasis	0.942	0.948	0.909
Wavelet-LHL_glrlm_LongRunLowGrayLevelEmphasis	0.639	0.665	0.545
Wavelet-LHL_glrlm_LowGrayLevelRunEmphasis	0.768	0.818	0.690
Wavelet-LHL_glrlm_RunEntropy	0.921	0.909	0.921
Wavelet-LHL_glrlm_RunLengthNonUniformity	0.997	0.997	0.904
Wavelet-LHL_glrlm_RunLengthNonUniformityNormalized	0.954	0.932	0.970
Wavelet-LHL_glrlm_RunPercentage	0.951	0.939	0.959
Wavelet-LHL_glrlm_RunVariance	0.926	0.931	0.865
Wavelet-LHL_glrlm_ShortRunEmphasis	0.951	0.942	0.958
Wavelet-LHL_glrlm_ShortRunHighGrayLevelEmphasis	0.943	0.948	0.939
Wavelet-LHL_glrlm_ShortRunLowGrayLevelEmphasis	0.724	0.742	0.693
Wavelet-LHL_glszm_GrayLevelNonUniformity	0.978	0.977	0.733
Wavelet-LHL_glszm_GrayLevelNonUniformityNormalized	0.866	0.886	0.825
Wavelet-LHL_glszm_GrayLevelVariance	0.902	0.956	0.865
Wavelet-LHL_glszm_HighGrayLevelZoneEmphasis	0.942	0.952	0.928
Wavelet-LHL_glszm_LargeAreaEmphasis	0.784	0.787	0.195
Wavelet-LHL_glszm_LargeAreaHighGrayLevelEmphasis	0.923	0.918	0.372
Wavelet-LHL_glszm_LargeAreaLowGrayLevelEmphasis	0.534	0.572	0.143
Wavelet-LHL_glszm_LowGrayLevelZoneEmphasis	0.804	0.746	0.874
Wavelet-LHL_glszm_SizeZoneNonUniformity	0.934	0.933	0.909
Wavelet-LHL_glszm_SizeZoneNonUniformityNormalized	0.784	0.704	0.817
Wavelet-LHL_glszm_SmallAreaEmphasis	0.740	0.651	0.801
Wavelet-LHL_glszm_SmallAreaHighGrayLevelEmphasis	0.936	0.953	0.916
Wavelet-LHL_glszm_SmallAreaLowGrayLevelEmphasis	0.024	0.094	0.041
Wavelet-LHL_glszm_ZoneEntropy	0.940	0.950	0.906
Wavelet-LHL_glszm_ZonePercentage	0.931	0.864	0.944
Wavelet-LHL_glszm_ZoneVariance	0.822	0.813	0.265
Wavelet-LHL_ngtdm_Busyness	0.757	0.782	0.049
Wavelet-LHL_ngtdm_Coarseness	0.960	0.896	0.953
Wavelet-LHL_ngtdm_Complexity	0.912	0.946	0.860
Wavelet-LHL_ngtdm_Contrast	0.797	0.933	0.780
Wavelet-LHL_ngtdm_Strength	0.875	0.940	0.857
Wavelet-LLH_firstorder_10Percentile	0.778	0.783	0.761
Wavelet-LLH_firstorder_90Percentile	0.666	0.569	0.707
Wavelet-LLH_firstorder_Energy	0.971	0.972	0.839
Wavelet-LLH_firstorder_Entropy	0.800	0.817	0.773
Wavelet-LLH_firstorder_InterquartileRange	0.710	0.608	0.733
Wavelet-LLH_firstorder_Kurtosis	0.584	0.489	0.515
Wavelet-LLH_firstorder_Maximum	0.844	0.876	0.764
Wavelet-LLH_firstorder_Mean	0.837	0.842	0.818
Wavelet-LLH_firstorder_MeanAbsoluteDeviation	0.740	0.703	0.744
Wavelet-LLH_firstorder_Median	0.699	0.647	0.683
Wavelet-LLH_firstorder_Minimum	0.820	0.848	0.778
Wavelet-LLH_firstorder_Range	0.835	0.864	0.781
Wavelet-LLH_firstorder_RobustMeanAbsoluteDeviation	0.724	0.655	0.734

Intraclass correlation coefficient (ICC)

0.1 0.2 0.3 0.4 0.5 0.6 0.7 0.8 0.9 1

Feature	All lesions	Large lesions	Small lesions
Wavelet-LLH_firstorder_RootMeanSquared	0.767	0.745	0.767
Wavelet-LLH_firstorder_Skewness	0.674	0.677	0.627
Wavelet-LLH_firstorder_TotalEnergy	0.961	0.961	0.842
Wavelet-LLH_firstorder_Uniformity	0.794	0.807	0.760
Wavelet-LLH_firstorder_Variance	0.742	0.571	0.786
Wavelet-LLH_glcm_Autocorrelation	0.850	0.902	0.700
Wavelet-LLH_glcm_ClusterProminence	0.725	0.613	0.733
Wavelet-LLH_glcm_ClusterShade	0.564	0.631	0.540
Wavelet-LLH_glcm_ClusterTendency	0.769	0.648	0.795
Wavelet-LLH_glcm_Contrast	0.748	0.396	0.816
Wavelet-LLH_glcm_Correlation	0.616	0.621	0.600
Wavelet-LLH_glcm_DifferenceAverage	0.752	0.650	0.776
Wavelet-LLH_glcm_DifferenceEntropy	0.788	0.783	0.784
Wavelet-LLH_glcm_DifferenceVariance	0.733	0.555	0.792
Wavelet-LLH_glcm_Id	0.796	0.796	0.766
Wavelet-LLH_glcm_Idm	0.797	0.796	0.768
Wavelet-LLH_glcm_Idmn	0.735	0.614	0.578
Wavelet-LLH_glcm_Idn	0.806	0.706	0.650
Wavelet-LLH_glcm_Imc1	0.826	0.845	0.734
Wavelet-LLH_glcm_Imc2	0.771	0.693	0.696
Wavelet-LLH_glcm_InverseVariance	0.709	0.686	0.686
Wavelet-LLH_glcm_JointAverage	0.821	0.852	0.756
Wavelet-LLH_glcm_JointEnergy	0.773	0.773	0.767
Wavelet-LLH_glcm_JointEntropy	0.814	0.825	0.797
Wavelet-LLH_glcm_MCC	0.785	0.664	0.746
Wavelet-LLH_glcm_MaximumProbability	0.754	0.761	0.733
Wavelet-LLH_glcm_SumAverage	0.821	0.852	0.756
Wavelet-LLH_glcm_SumEntropy	0.804	0.825	0.772
Wavelet-LLH_glcm_SumSquares	0.765	0.566	0.807
Wavelet-LLH_glrlm_GrayLevelNonUniformity	0.922	0.926	0.518
Wavelet-LLH_glrlm_GrayLevelNonUniformityNormalized	0.807	0.829	0.765
Wavelet-LLH_glrlm_GrayLevelVariance	0.744	0.587	0.786
Wavelet-LLH_glrlm_HighGrayLevelRunEmphasis	0.838	0.889	0.728
Wavelet-LLH_glrlm_LongRunEmphasis	0.783	0.801	0.734
Wavelet-LLH_glrlm_LongRunHighGrayLevelEmphasis	0.844	0.873	0.729
Wavelet-LLH_glrlm_LongRunLowGrayLevelEmphasis	0.506	0.434	0.779
Wavelet-LLH_glrlm_LowGrayLevelRunEmphasis	0.558	0.541	0.561
Wavelet-LLH_glrlm_RunEntropy	0.832	0.849	0.811
Wavelet-LLH_glrlm_RunLengthNonUniformity	0.993	0.993	0.914
Wavelet-LLH_glrlm_RunLengthNonUniformityNormalized	0.810	0.805	0.776
Wavelet-LLH_glrlm_RunPercentage	0.831	0.824	0.804
Wavelet-LLH_glrlm_RunVariance	0.763	0.776	0.712
Wavelet-LLH_glrlm_ShortRunEmphasis	0.834	0.840	0.800
Wavelet-LLH_glrlm_ShortRunHighGrayLevelEmphasis	0.836	0.890	0.728
Wavelet-LLH_glrlm_ShortRunLowGrayLevelEmphasis	0.568	0.546	0.544
Wavelet-LLH_glszm_GrayLevelNonUniformity	0.982	0.982	0.764
Wavelet-LLH_glszm_GrayLevelNonUniformityNormalized	0.763	0.748	0.783
Wavelet-LLH_glszm_GrayLevelVariance	0.767	0.762	0.770
Wavelet-LLH_glszm_HighGrayLevelZoneEmphasis	0.844	0.903	0.720
Wavelet-LLH_glszm_LargeAreaEmphasis	0.425	0.455	0.164
Wavelet-LLH_glszm_LargeAreaHighGrayLevelEmphasis	0.761	0.761	0.120
Wavelet-LLH_glszm_LargeAreaLowGrayLevelEmphasis	0.169	0.159	0.210
Wavelet-LLH_glszm_LowGrayLevelZoneEmphasis	0.631	0.643	0.616
Wavelet-LLH_glszm_SizeZoneNonUniformity	0.970	0.971	0.791
Wavelet-LLH_glszm_SizeZoneNonUniformityNormalized	0.808	0.794	0.804
Wavelet-LLH_glszm_SmallAreaEmphasis	0.780	0.805	0.759
Wavelet-LLH_glszm_SmallAreaHighGrayLevelEmphasis	0.827	0.917	0.697
Wavelet-LLH_glszm_SmallAreaLowGrayLevelEmphasis	0.496	0.524	0.466
Wavelet-LLH_glszm_ZoneEntropy	0.871	0.886	0.813
Wavelet-LLH_glszm_ZonePercentage	0.820	0.720	0.823
Wavelet-LLH_glszm_ZoneVariance	0.413	0.435	0.131
Wavelet-LLH_ngtdm_Busyness	0.437	0.405	0.501
Wavelet-LLH_ngtdm_Coarseness	0.971	0.901	0.969
Wavelet-LLH_ngtdm_Complexity	0.819	0.846	0.797
Wavelet-LLH_ngtdm_Contrast	0.644	0.312	0.661
Wavelet-LLH_ngtdm_Strength	0.858	0.735	0.847
Wavelet-LLL_firstorder_10Percentile	0.979	0.987	0.962
Wavelet-LLL_firstorder_90Percentile	0.972	0.985	0.945
Wavelet-LLL_firstorder_Energy	0.999	0.999	0.991
Wavelet-LLL_firstorder_Entropy	0.959	0.971	0.938
Wavelet-LLL_firstorder_InterquartileRange	0.946	0.966	0.911
Wavelet-LLL_firstorder_Kurtosis	0.878	0.891	0.826
Wavelet-LLL_firstorder_Maximum	0.976	0.987	0.951
Wavelet-LLL_firstorder_Mean	0.976	0.986	0.954
Wavelet-LLL_firstorder_MeanAbsoluteDeviation	0.953	0.973	0.919
Wavelet-LLL_firstorder_Median	0.975	0.985	0.952
Wavelet-LLL_firstorder_Minimum	0.962	0.959	0.965
Wavelet-LLL_firstorder_Range	0.964	0.974	0.934
Wavelet-LLL_firstorder_RobustMeanAbsoluteDeviation	0.950	0.970	0.916
Wavelet-LLL_firstorder_RootMeanSquared	0.975	0.986	0.953
Wavelet-LLL_firstorder_Skewness	0.794	0.834	0.643
Wavelet-LLL_firstorder_TotalEnergy	1.000	1.000	0.987
Wavelet-LLL_firstorder_Uniformity	0.956	0.972	0.939
Wavelet-LLL_firstorder_Variance	0.968	0.982	0.940
Wavelet-LLL_glcm_Autocorrelation	0.946	0.948	0.928
Wavelet-LLL_glcm_ClusterProminence	0.989	0.990	0.983
Wavelet-LLL_glcm_ClusterShade	0.973	0.981	0.931
Wavelet-LLL_glcm_ClusterTendency	0.973	0.984	0.946
Wavelet-LLL_glcm_Contrast	0.918	0.929	0.912
Wavelet-LLL_glcm_Correlation	0.922	0.847	0.925
Wavelet-LLL_glcm_DifferenceAverage	0.908	0.917	0.899
Wavelet-LLL_glcm_DifferenceEntropy	0.910	0.935	0.878
Wavelet-LLL_glcm_DifferenceVariance	0.915	0.939	0.901
Wavelet-LLL_glcm_Id	0.904	0.928	0.879
Wavelet-LLL_glcm_Idm	0.894	0.928	0.860
Wavelet-LLL_glcm_Idmn	0.924	0.867	0.905
Wavelet-LLL_glcm_Idn	0.922	0.875	0.899
Wavelet-LLL_glcm_Imc1	0.933	0.817	0.937
Wavelet-LLL_glcm_Imc2	0.889	0.885	0.885
Wavelet-LLL_glcm_InverseVariance	0.897	0.919	0.872
Wavelet-LLL_glcm_JointAverage	0.930	0.935	0.907
Wavelet-LLL_glcm_JointEnergy	0.914	0.970	0.869
Wavelet-LLL_glcm_JointEntropy	0.965	0.953	0.959
Wavelet-LLL_glcm_MCC	0.852	0.814	0.864
Wavelet-LLL_glcm_MaximumProbability	0.933	0.969	0.875
Wavelet-LLL_glcm_SumAverage	0.930	0.935	0.907
Wavelet-LLL_glcm_SumEntropy	0.966	0.972	0.949
Wavelet-LLL_glcm_SumSquares	0.965	0.981	0.937
Wavelet-LLL_glrlm_GrayLevelNonUniformity	0.910	0.936	0.621
Wavelet-LLL_glrlm_GrayLevelNonUniformityNormalized	0.954	0.970	0.939
Wavelet-LLL_glrlm_GrayLevelVariance	0.969	0.983	0.940
Wavelet-LLL_glrlm_HighGrayLevelRunEmphasis	0.948	0.949	0.929
Wavelet-LLL_glrlm_LongRunEmphasis	0.723	0.907	0.627
Wavelet-LLL_glrlm_LongRunHighGrayLevelEmphasis	0.945	0.945	0.926
Wavelet-LLL_glrlm_LongRunLowGrayLevelEmphasis	0.719	0.767	0.670
Wavelet-LLL_glrlm_LowGrayLevelRunEmphasis	0.725	0.734	0.694
Wavelet-LLL_glrlm_RunEntropy	0.966	0.974	0.945
Wavelet-LLL_glrlm_RunLengthNonUniformity	0.994	0.994	0.841
Wavelet-LLL_glrlm_RunLengthNonUniformityNormalized	0.869	0.925	0.816
Wavelet-LLL_glrlm_RunPercentage	0.853	0.924	0.785
Wavelet-LLL_glrlm_RunVariance	0.718	0.898	0.613
Wavelet-LLL_glrlm_ShortRunEmphasis	0.838	0.924	0.771
Wavelet-LLL_glrlm_ShortRunHighGrayLevelEmphasis	0.948	0.949	0.930
Wavelet-LLL_glrlm_ShortRunLowGrayLevelEmphasis	0.729	0.726	0.703
Wavelet-LLL_glszm_GrayLevelNonUniformity	0.986	0.986	0.870
Wavelet-LLL_glszm_GrayLevelNonUniformityNormalized	0.679	0.867	0.598
Wavelet-LLL_glszm_GrayLevelVariance	0.970	0.984	0.939
Wavelet-LLL_glszm_HighGrayLevelZoneEmphasis	0.944	0.945	0.929
Wavelet-LLL_glszm_LargeAreaEmphasis	0.164	0.192	0.156
Wavelet-LLL_glszm_LargeAreaHighGrayLevelEmphasis	0.137	0.246	0.089
Wavelet-LLL_glszm_LargeAreaLowGrayLevelEmphasis	0.141	0.152	0.134
Wavelet-LLL_glszm_LowGrayLevelZoneEmphasis	0.492	0.626	0.405
Wavelet-LLL_glszm_SizeZoneNonUniformity	0.995	0.995	0.948
Wavelet-LLL_glszm_SizeZoneNonUniformityNormalized	0.898	0.910	0.885
Wavelet-LLL_glszm_SmallAreaEmphasis	0.889	0.899	0.877
Wavelet-LLL_glszm_SmallAreaHighGrayLevelEmphasis	0.947	0.951	0.927
Wavelet-LLL_glszm_SmallAreaLowGrayLevelEmphasis	0.396	0.603	0.294
Wavelet-LLL_glszm_ZoneEntropy	0.927	0.947	0.863
Wavelet-LLL_glszm_ZonePercentage	0.911	0.920	0.895
Wavelet-LLL_glszm_ZoneVariance	0.153	0.188	0.142
Wavelet-LLL_ngtdm_Busyness	0.785	0.819	0.742
Wavelet-LLL_ngtdm_Coarseness	0.964	0.940	0.957
Wavelet-LLL_ngtdm_Complexity	0.972	0.978	0.960
Wavelet-LLL_ngtdm_Contrast	0.836	0.860	0.821
Wavelet-LLL_ngtdm_Strength	0.948	0.942	0.945

FIGURE S1 (continued).

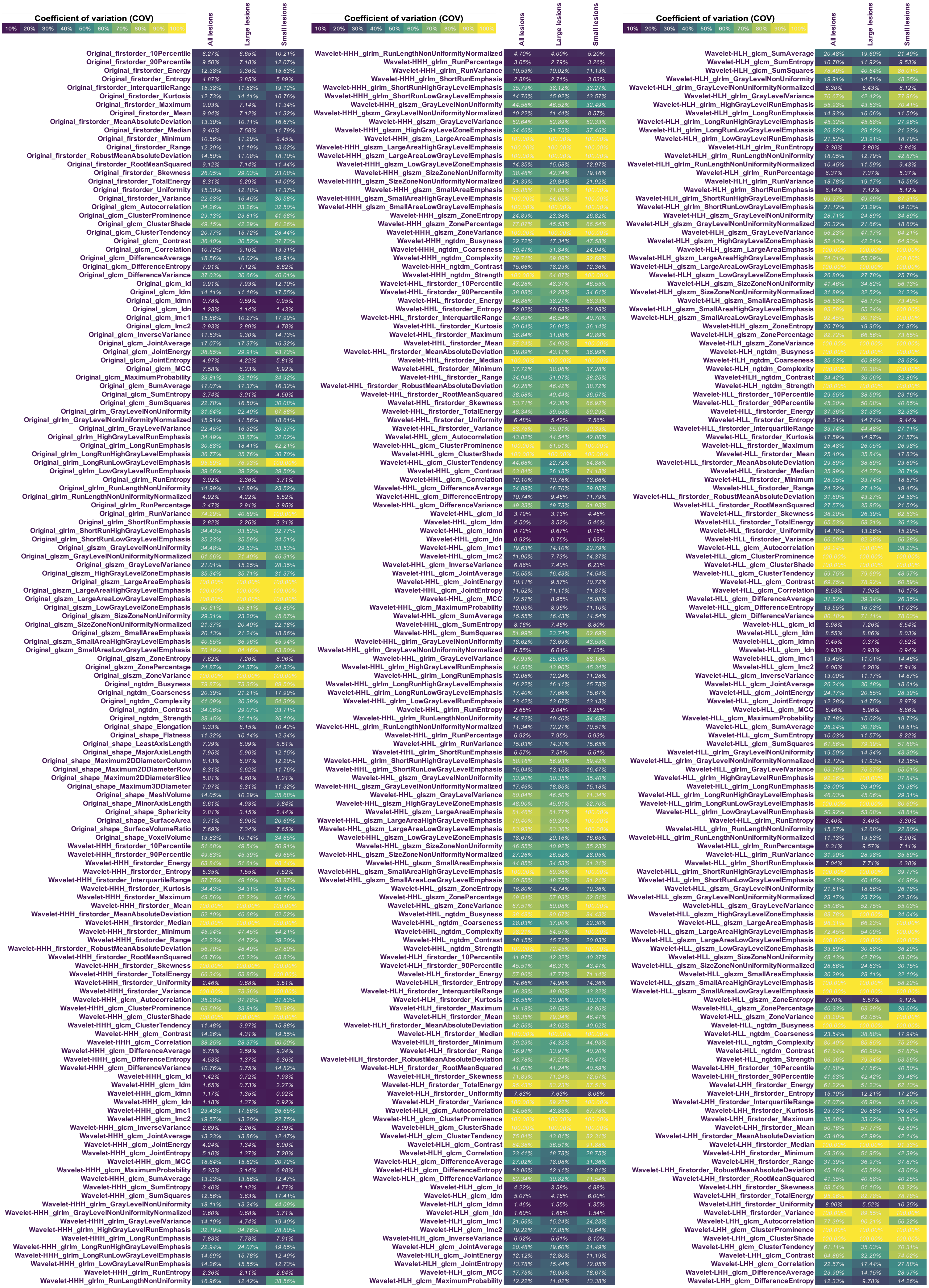

FIGURE S2 Heatmap of radiomic features based on coefficient of variation (COV) divided by lesion size.

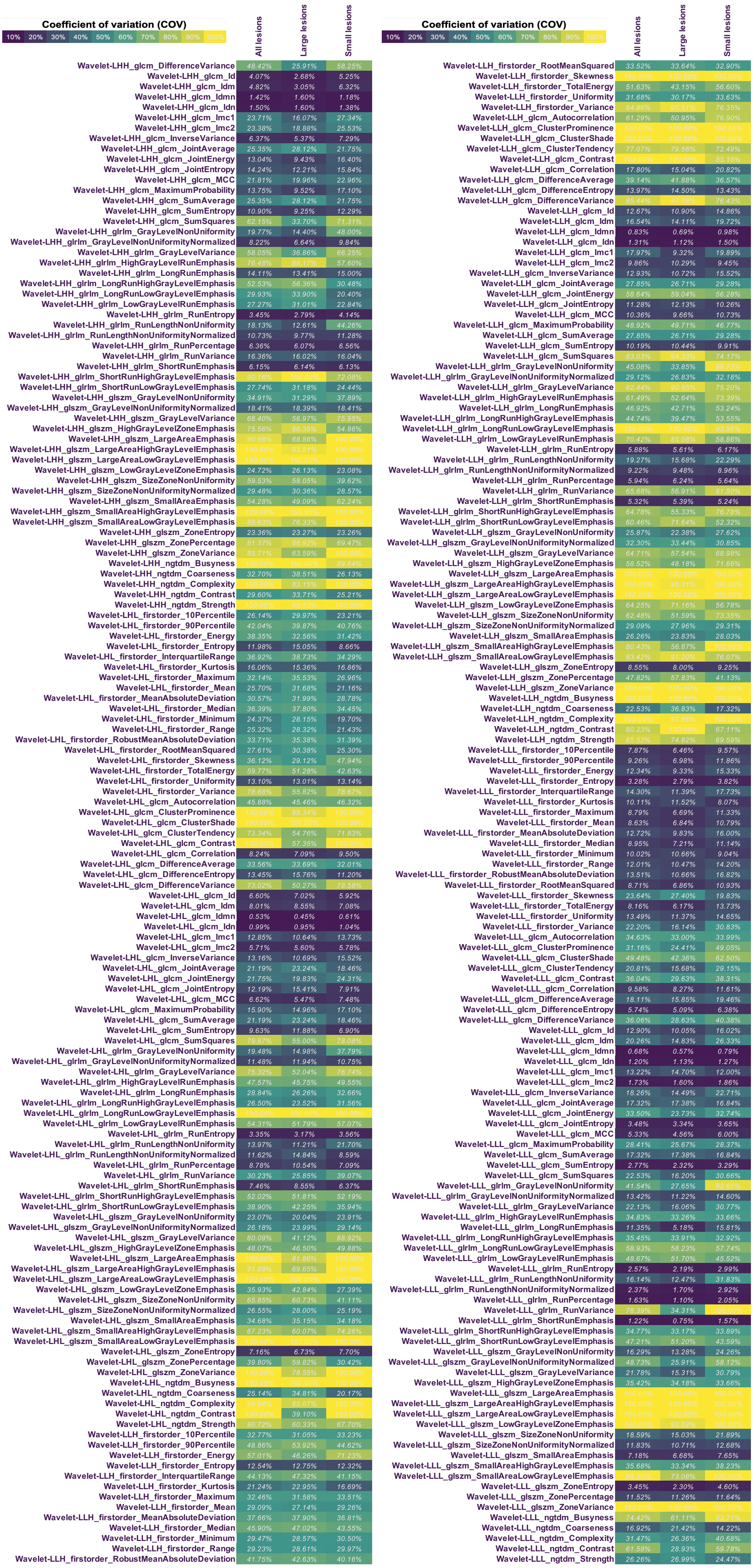

FIGURE S2 (continued).
