# Supplementary material for: Impact of respiratory motion on 18F‐FDG PET radiomics stability: Clinical evaluation with a digital PET scanner
Source: J Appl Clin Med Phys. 2023 Nov 8;24(12):e14200. doi: 10.1002/acm2.14200 (PMC10691638; doi:10.1002/acm2.14200)
Supplement: Supplementary file 2 — Supporting‐Information [file ACM2-24-e14200-s002.pdf]

TABLE S1 List of radiomic features (n = 725)

Type	Description
First-order (n = 18)	10 Percentile, 90 Percentile, Energy, Entropy, Interquartile Range, Kurtosis, Maximum, Mean, Mean Absolute Deviation, Median, Minimum, Range, Robust Mean Absolute Deviation, Root Mean Squared, Skewness, Total Energy, Uniformity, Variance
Shape (n = 14)	Elongation, Flatness, Least Axis Length, Major Axis Length, Maximum 2D Diameter Column, Maximum 2D Diameter Row, Maximum 2D Diameter Slice, Maximum 3D Diameter, Mesh Volume, Minor Axis Length, Sphericity, Surface Area, Surface Volume Ratio, Voxel Volume
GLCM (n = 24)	Autocorrelation, Cluster Prominence, Cluster Shade, Cluster Tendency, Contrast, Correlation, Difference Average, Difference Entropy, Difference Variance, Inverse Difference, Inverse Difference Moment, Inverse Difference Moment Normalized, Inverse Difference Normalized, Informational Measure of Correlation 1, Informational Measure of Correlation 2, Inverse Variance, Joint Average, Joint Energy, Joint Entropy, Maximal Correlation Coefficient, Maximum Probability, Sum Average, Sum Entropy, Sum Squares
GLRLM (n = 16)	Gray Level NonUniformity, Gray Level NonUniformity Normalized, Gray Level Variance, High Gray Level Run Emphasis, Long Run Emphasis, Long Run High Gray Level Emphasis, Long Run Low Gray Level Emphasis, Low Gray Level Run Emphasis, Run Entropy, Run Length NonUniformity, Run Length NonUniformity Normalized, Run Percentage, Run Variance, Short Run Emphasis, Short Run High Gray Level Emphasis, Short Run Low Gray Level Emphasis
GLSZM (n = 16)	Gray Level NonUniformity, Gray Level NonUniformity Normalized, Gray Level Variance, High Gray Level Zone Emphasis, Large Area Emphasis, Large Area High Gray Level Emphasis, Large Area Low Gray Level Emphasis, Low Gray Level Zone Emphasis, Size Zone NonUniformity, Size Zone NonUniformity Normalized, Small Area Emphasis, Small Area High Gray Level Emphasis, Small Area Low Gray Level Emphasis, Zone Entropy, Zone Percentage, Zone Variance
NGTDM (n = 5)	Busyness, Coarseness, Complexity, Contrast, Strength
Wavelet_HHH (n = 79)<br>Wavelet_HHL (n = 79)<br>Wavelet_HLH (n = 79)<br>Wavelet_HLL (n = 79)<br>Wavelet_LHH (n = 79)<br>Wavelet_LHL (n = 79)<br>Wavelet_LLH (n = 79)<br>Wavelet_LLL (n = 79)	First-order_10 Percentile, First-order_90 Percentile, First-order_Energy, First-order_Entropy, First-order_Interquartile Range, First-order_Kurtosis, First-order_Maximum, First-order_Mean, First-order_Mean Absolute Deviation, First-order_Median, First-order_Minimum, First-order_Range, First-order_Robust Mean Absolute Deviation, First-order_Root Mean Squared, First-order_Skewness, First-order_Total Energy, First-

	order_Uniformity, First-order_Variance, GLCM_Autocorrelation, GLCM_Cluster Prominence, GLCM_Cluster Shade, GLCM_Cluster Tendency, GLCM_Contrast, GLCM_Correlation, GLCM_Difference Average, GLCM_Difference Entropy, GLCM_Difference Variance, GLCM_Inverse Difference, GLCM_Inverse Difference Moment, GLCM_Inverse Difference Moment Normalized, GLCM_Inverse Difference Normalized, GLCM_Informational Measure of Correlation 1, GLCM_Informational Measure of Correlation 2, GLCM_Inverse Variance, GLCM_Joint Average, GLCM_Joint Energy, GLCM_Joint Entropy, GLCM_Maximal Correlation Coefficient, GLCM_Maximum Probability, GLCM_Sum Average, GLCM_Sum Entropy, GLCM_Sum Squares, GLRLM_Gray Level NonUniformity, GLRLM_Gray Level NonUniformity Normalized, GLRLM_Gray Level Variance, GLRLM_High Gray Level Run Emphasis, GLRLM_Long Run Emphasis, GLRLM_Long Run High Gray Level Emphasis, GLRLM_Long Run Low Gray Level Emphasis, GLRLM_Low Gray Level Run Emphasis, GLRLM_Run Entropy, GLRLM_Run Length NonUniformity, GLRLM_Run Length NonUniformity Normalized, GLRLM_Run Percentage, GLRLM_Run Variance, GLRLM_Short Run Emphasis, GLRLM_Short Run High Gray Level Emphasis, GLRLM_Short Run Low Gray Level Emphasis, GLSZM_Gray Level NonUniformity, GLSZM_Gray Level NonUniformity Normalized, GLSZM_Gray Level Variance, GLSZM_High Gray Level Zone Emphasis, GLSZM_Large Area Emphasis, GLSZM_Large Area High Gray Level Emphasis, GLSZM_Large Area Low Gray Level Emphasis, GLSZM_Low Gray Level Zone Emphasis, GLSZM_Size Zone NonUniformity, GLSZM_Size Zone NonUniformity Normalized, GLSZM_Small Area Emphasis, GLSZM_Small Area High Gray Level Emphasis, GLSZM_Small Area Low Gray Level Emphasis, GLSZM_Zone Entropy, GLSZM_Zone Percentage, GLSZM_Zone Variance, NGTDM_Busyness, NGTDM_Coarseness, NGTDM_Complexity, NGTDM_Contrast, NGTDM_Strength

GLCM, gray-level co-occurrence matrix; GLRLM, gray-level run-length matrix; GLSZM, gray-level size-zone matrix; NGTDM, neighboring gray-tone difference matrix. Wavelet filtering yielded eight decompositions, applying either a High (H) or a Low (L) pass filter in each x-, y-, and z-dimension.

TABLE S2 Radiomic features with high and low stabilities against respiratory motion in lesions with metabolic tumor volumes ≥ 3 cm^3

Group	Type	Description
High stability	First-order	Entropy
(ICC ≥ 0.9 and COV ≤ 5%)	Shape	Maximum 2D Diameter Slice
		Minor Axis Length
		Sphericity
	GLCM	Joint Entropy
		Sum Entropy
	GLRLM	Run Entropy
		Run Length Non Uniformity Normalized
		Run Percentage
		Short Run Emphasis
	Wavelet	HHH_First-order_Entropy
		HHH_First-order_Uniformity
		HHH_GLCM_Cluster Tendency
		HHH_GLCM_Contrast
		HHH_GLCM_Difference Average
		HHH_GLCM_Difference Entropy
		HHH_GLCM_Difference Variance
		HHH_GLCM_Inverse Difference Moment
		HHH_GLCM_Joint Energy
		HHH_GLCM_Joint Entropy
		HHH_GLCM_Sum Entropy
		HHH_GLCM_Sum Squares
		HHH_GLRLM_Gray Level NonUniformity Normalized
		HHH_GLRLM_Gray Level Variance
		HHH_GLRLM_Run Entropy
		HHH_GLRLM_Run Percentage
		HHH_GLRLM_Short Run Emphasis
		HHL_GLRLM_Run Entropy
		LHL_GLCM_Inverse Difference Normalized
		LHL_GLRLM_Run Entropy
		LLL_First-order_Entropy
		LLL_GLCM_Joint Entropy
		LLL_GLCM_Sum Entropy
		LLL_GLRLM_Run Entropy
		LLL_GLRLM_Run Length NonUniformity Normalized
		LLL_GLRLM_Run Percentage
		LLL_GLRLM_Short Run Emphasis
		LLL_GLSZM_Zone Entropy
Low stability	GLSZM	Large Area Emphasis
(ICC < 0.5 and COV > 20%)		Large Area High Gray Level Emphasis
		Large Area Low Gray Level Emphasis
		Small Area Low Gray Level Emphasis
		ZoneVariance
	Wavelet	HHH_First-order_Median
		HHH_GLSZM_Size Zone Non Uniformity Normalized

	HHH_GLSZM_Small Area LowG ray Level Emphasis
	HHL_GLSZM_Size Zone Non Uniformity Normalized
	HHL_GLSZM_Small Area Emphasis
	HLH_First-order_Median
	HLH_First-order_Skewness
	HLH_GLSZM_Size Zone Non Uniformity Normalized
	HLH_GLSZM_Small Area Emphasis
	HLH_GLSZM_SmallArea Low Gray Level Emphasis
	HLL_GLSZM_Small Area Low Gray Level Emphasis
	HLL_NGTDM_Busyness
	LHH_First-order_Median
	LHH_GLSZM_Size Zone Non Uniformity Normalized
	LHH_GLSZM_Small Area Emphasis
	LHH_GLSZM_Small Area Low Gray Level Emphasis
	LHL_GLSZM_Small Area Low Gray Level Emphasis
	LLH_First-order_Kurtosis
	LLH_GLCM_Contrast
	LLH_GLRLM_Long Run Low Gray Level Emphasis
	LLH_GLSZM_Large Area Emphasis
	LLH_GLSZM_Large Area Low Gray Level Emphasis
	LLH_GLSZM_Zone Variance
	LLH_NGTDM_Busyness
	LLH_NGTDM_Contrast
	LLL_GLSZM_Large Area Emphasis
	LLL_GLSZM_Large Area High Gray Level Emphasis
	LLL_GLSZM_Large Area Low Gray Level Emphasis
	LLL_GLSZM_Zone Variance

ICC, intraclass correlation coefficient; COV, coefficient of variation; GLCM, gray-level co-occurrence matrix; GLRLM, gray-level run-length matrix; GLSZM, gray-level size-zone matrix; NGTDM, neighboring gray-tone difference matrix. Wavelet filtering yielded eight decompositions, applying either a High (H) or a Low (L) pass filter in each of the x-, y-, and z-dimensions.

TABLE S3 Radiomic features with high and low stabilities against respiratory motion in lesions with metabolic tumor volumes < 3 cm^3

Group	Type	Description
High stability (ICC ≥ 0.9 and COV ≤ 5%)	Shape	Sphericity
	GLCM	Sum Entropy
	GLRLM	Run Entropy
	Wavelet	HHH_GLRLM_Run Entropy
		HHH_GLRLM_Run Percentage
		HHH_GLRLM_Short Run Emphasis
		HHL_GLCM_Inverse Difference
		HLL_GLCM_Inverse Difference Moment Normalized
		HLL_GLCM_Inverse Difference Normalized
		HLL_GLRLM_Run Entropy
		LHL_GLCM_Inverse Difference Normalized
		LHL_GLRLM_Run Entropy
		LLL_First-order_Entropy
		LLL_GLCM_Inverse Difference Moment Normalized
		LLL_GLCM_Joint Entropy
		LLL_GLCM_Sum Entropy
		LLL_GLRLM_Run Entropy
Low stability (ICC < 0.5 and COV > 20%)	Shape	Mesh Volume
		Surface Area
		Voxel Volume
	GLSZM	Large Area Emphasis
		Large Area High Gray Level Emphasis
		Large Area Low Gray Level Emphasis
		Small Area Low Gray Level Emphasis
		ZoneVariance
	Wavelet	HHH_First-order_Skewness
		HHH_GLCM_Cluster Shade
		HHH_GLSZM_Gray Level Non Uniformity
		HHH_GLSZM_Large Area Emphasis
		HHH_GLSZM_Large Area High Gray Level Emphasis
		HHH_GLSZM_Large Area Low Gray Level Emphasis
		HHH_GLSZM_Size Zone Non Uniformity Normalized
		HHH_GLSZM_Small Area Emphasis
		HHH_GLSZM_Small Area Low Gray Level Emphasis
		HHH_GLSZM_Zone Entropy
		HHH_GLSZM_Zone Variance
		HHL_GLSZM_Large Area Emphasis
		HHL_GLSZM_Large Area High Gray Level Emphasis
		HHL_GLSZM_Large Area Low Gray Level Emphasis
		HHL_GLSZM_Size Zone Non Uniformity Normalized
		HHL_GLSZM_Small Area Emphasis
		HHL_GLSZM_Small Area Low Gray Level Emphasis
		HHL_GLSZM_Zone Variance
		HLH_GLCM_Cluster Shade
		HLH_GLSZM_Gray Level NonUniformity

	HLH_GLSZM_Large Area Emphasis
	HLH_GLSZM_Large Area High Gray Level Emphasis
	HLH_GLSZM_Large Area Low Gray Level Emphasis
	HLH_GLSZM_Size Zone Non Uniformity
	HLH_GLSZM_Size Zone Non Uniformity Normalized
	HLH_GLSZM_Small Area Emphasis
	HLH_GLSZM_Small Area Low Gray Level Emphasis
	HLH_GLSZM_Zone Variance
	HLH_NGTDM_Busyness

ICC, intraclass correlation coefficient; COV, coefficient of variation; GLCM, gray-level co-occurrence matrix; GLRLM, gray-level run-length matrix; GLSZM, gray-level size-zone matrix; NGTDM, neighboring gray-tone difference matrix. Wavelet filtering yielded eight decompositions, applying either a High (H) or a Low (L) pass filter in each x-, y-, and z-dimension.
